# Universal Prevention of Dementia in Italy: A Document Analysis of the 21 Italian Regional Prevention Plans

**DOI:** 10.14283/jpad.2024.144

**Published:** 2024-07-11

**Authors:** S. Salemme, D. Marconi, S. M. Pani, G. Zamboni, C. Sardu, G. Lazzeri, M. Corbo, E. Lacorte, N. Locuratolo, A. Ancidoni, N. Vanacore, Guido Bellomo

**Affiliations:** 1 Department of Biomedical, Metabolic and Neural Sciences, University of Modena and Reggio Emilia, Modena, Italy; 2 International School of Advanced Studies, University of Camerino, Camerino, Italy; 3 Post Graduate School of Public Health, University of Siena, Siena, Italy; 4 Department of Medical Sciences and Public Health, University of Cagliari–Cittadella Universitaria Monserrato, Monserrato, CA Italy; 5 Neurology Unit, Baggiovara Hospital, Azienda Ospedaliero Universitaria di Modena, Modena, Italy; 6 Department of Molecular and Developmental Medicine, University of Siena, Siena, Italy; 7 Department of Neurorehabilitation Sciences, Casa di Cura Igea, Milan, Italy; 8 National Center For Disease Prevention and Health Promotion, National Institute of Health, Rome, Italy Via Giano della Bella 34, 00161

**Keywords:** Dementia, prevention, population approach, risk factors, health policy

## Abstract

**Background:**

Up to 40% of dementia cases are theoretically avoidable and population-level interventions (i.e., universal prevention) are a key component in facing the global public health challenge of dementia. However, information on the agenda for the universal prevention of dementia at the national and sub-national levels is still lacking.

**Objectives:**

We aim to provide a comprehensive description of the universal prevention strategies specific to dementia in Italian regions and autonomous provinces (APs).

**Design:**

We conducted a document analysis of the 21 Italian Regional Prevention Plans (RPPs), with a focus on interventions that target potentially modifiable risk factors for dementia. We analysed the final version of the documents, which were previously downloaded from the dedicated section of the Italian Ministry of Health website in January 2023. We classified the interventions as direct, indirect, or absent. Additionally, we created a quality checklist to outline the essential programmatic elements and applied it to summarise the key findings of the RPPs.

**Measurements:**

We reported the number of populationlevel interventions specific for dementia with sub-national detail. We reported information on the risk factor targeted by the interventions, the age groups and populations they were designed for. We summarized the presence or absence of 63 programmatic items using a four-domain checklist.

**Results:**

We identified 248 interventions for dementia prevention among the assessed RPPs: 100% of the plans addressed physical inactivity; 30–35% addressed smoking, alcohol, obesity, and social isolation; 25% addressed hypertension, diabetes, and air pollution; only 5–10% addressed education, depression, and hearing loss. Most interventions targeted the general population. Quality checklist scores significantly varied among regions, with demographics and prevention strategies domains scoring higher than disease burden and intervention feasibility ones.

**Conclusions:**

The population-level interventions in the Italian Regional Prevention Programs dedicated to dementia prevention primarily focus on vascular risk factors, with limited coverage of dementia-specific factors such as traumatic brain injury and hearing loss. This data should be considered when planning future interventions for dementia prevention.

**Electronic Supplementary Material:**

Supplementary material is available in the online version of this article at 10.14283/jpad.2024.144.

## Introduction

**D**ementia is one of the major public health challenges that national health systems are currently facing. Besides being the seventh leading cause of death worldwide, dementia is also responsible for a significant share of disability-adjusted life years (DALYs): this share increased to 28,352 in the years 2000 to 2019, reflecting a 122% increase (1). The ageing and population increase are expected to further affect this challenge’s extent and complexity. In 2019, people aged 60 and over were estimated to be about 1 billion. By 2030, they are expected to increase up to 1.4 billion and exceed 2.1 billion by 2050 (2). Epidemiological data and cost estimates highlight the need to address the increasing impact of dementia by adopting a universal prevention approach aimed at reducing the frequency of potentially modifiable risk factors through interventions targeted to the general population (3, 4). According to the Lancet Commission on Dementia Prevention, Intervention, and Care, up to 40% of dementia cases globally are theoretically avoidable. Specifically, the commission identified 12 potentially modifiable risk factors: lower education, hypertension, hearing loss, smoking, obesity, depression, physical inactivity, diabetes, social isolation, excessive alcohol consumption, traumatic brain injury and air pollution. Risk factors intervene throughout an individual’s life span, contributing from early life to old age to increasing the risk of cognitive decline (5). The implementation of national preventive strategies for dementia is one of the targets of the Global Action Plan on the public health response to dementia launched by the World Health Organization (WHO) in 2017 (6). The WHO further defined action areas by publishing guidelines on evidence-based interventions for the primary and secondary prevention of dementia (7). However, the number of implemented preventive population approaches is small, as shown by the lack of operational national dementia plans and the limited amount of dedicated funds (8–10). Approved and signed in December 2021, the Italian Fund for Alzheimer’s and other dementias (IFAD) received 15 million euros for funding its 2021–2023 activities aimed at improving the quality of care for dementia from a public health perspective. The Dementia Observatory of the Italian National Institute of Health carried out eight activities within the IFAD. These included developing a national guideline for the diagnosis and treatment of dementia and Mild Cognitive Impairment (MCI), updating and implementing the National Dementia Plan (NDP), providing training and support activities for healthcare professionals and caregivers, and promoting strategies and actions for the primary and secondary prevention of dementia (Activity 5) (11). Within this framework, our research aimed to describe population approaches for the prevention of dementia (i.e., universal prevention) in Italy at a national and local level. To this purpose, we critically reviewed the 2020–2025 National Prevention Plan (NPP) and analysed the 21 Regional Prevention Plans (RPPs) (12, 13). The NPP serves as a valuable programming, monitoring, and evaluation system for implementing interventions aimed at collective prevention and public health. Within this framework, we have categorised all the interventions outlined in the context of the NPP and its subnational adaptations, the RPPs, as populationlevel interventions. The Italian National Health Service has undergone gradual decentralisation over the past three decades, with health powers being transferred to the regions and autonomous provinces. These entities have legislative power within their territories on matters not reserved to the Parliament and are responsible for meeting healthcare objectives set by the central state. However, this decentralisation has led to significant variability in health administration and service delivery across the country. To provide a comprehensive overview of the universal prevention strategies specific to dementia in Italy, we analysed the RPPs, which are the local adaptation of the NPP in each of the Italian regions and autonomous provinces. These documents outline specific actions aimed at meeting the health needs of the local population, are enacted periodically, must comply with specific structure requirements, and are monitored at the central level to provide an overall picture of the commitment to preventing dementia.

We propose an approach for identifying and critically reporting population-level interventions to prevent dementia while presenting a summary of the Italian scenario, where dedicated documents are defined centrally and adapted locally.

## Methods

### National and Regional Prevention Plans

**Figure 1:**
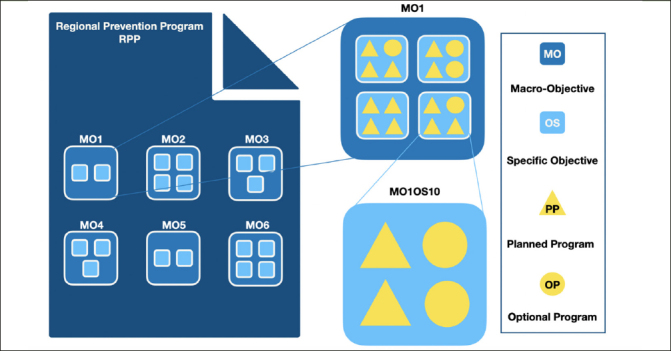
Overall architecture of Regional Prevention Programs MO1: Chronic non-communicable diseases; MO2: Addictions and related problems; MO3: Road and domestic accidents; MO4: Work accidents and injuries and occupational diseases; MO5: Environment, climate and health; MO6: Priority infectious diseases; MO1OS10: Development of a proactive management of modifiable dementia risk factors to delay or slow the onset or progression of the disease.

The NPP is a national document including all disease prevention and health promotion interventions. It includes six Macro Objectives (MOs): MO1 - Chronic non-communicable diseases (NCDs), MO2 - Addictions and related problems, MO3 - Road and domestic accidents, MO4 - Work accidents and injuries and occupational diseases, MO5 - Environment, climate, and health and MO6 - Priority infectious diseases. Each of these Macro Objectives includes Specific Objectives (SOs). Dementia is listed within the Specific Objective 10 of the Macro Objective 1 (MO1OS10): “Development of a proactive management of modifiable dementia risk factors to delay or slow the onset or progression of the disease” (12). Each Italian region (n=20) and autonomous province (AP; n=2) is required to adopt the NPP and adapt it to the local context by defining and approving an RPP. To this purpose, regions and autonomous provinces define interventions targeted to the Planned Programs (PPs) objectives outlined in the NPP. They can also identify additional programs, indicated as Optional Programs (OPs), to address Specific Objectives only partially covered by the Planned Programs (see Figure 1). We focused on the most recently published RPPs for the years 2020–2025, and carried out a document analysis based on document preparation, data extraction and analysis, and reporting of results according to the READ approach (Ready materials, Extract data, Analyse data, and Distil findings) (14).

### Search strategy

By October 2021, the Ministry of Health reviewed the RPPs drafted by the Italian regions and autonomous provinces. The final version of the documents, officially adopted by the regions and autonomous provinces by December 2021, was uploaded and made publicly available in a dedicated section of the Italian Ministry of Health website (13). We accessed the website in January 2023 and downloaded the RPPs of all regions and autonomous provinces for consultation and analysis.

### Identification of dementia-specific preventive interventions

We initially searched for preventive interventions specifically aiming at identifying interventions for dementia prevention within the RPPs. PPs and OPs related to MO1OS10 were identified using a synoptic table in each RPP. For each PP and OP, we analysed the full text of dementia prevention interventions, extracting useful information for defining target (i) risk factors, (ii) age groups, and (iii) populations. Our current study is part of a larger project that aims to integrate data on prevention planning with epidemiological data on the distribution of risk factors and modelling data on the proportion of avoidable cases of dementia. We adopted as a reference the potentially modifiable risk factors outlined by the Lancet Commission as the related population-attributable fractions were estimated and available. Using as a reference the list of potentially modifiable risk factors for dementia provided by the Lancet Commission, we assessed for the presence or absence of preventive interventions addressing each risk factor. We categorised as direct all interventions that explicitly addressed a risk factor. Interventions that could indirectly affect other risk factors were categorised as potential. In the absence of interventions directly or indirectly addressing a specific risk factor, we reported the category as absent. Interventions were defined as potentially having indirect effects based on a structured review (i.e., meta-analysis or systematic reviews) of published literature on plausible relationships between risk factors (Supplementary Table 1). Interventions that addressed lifestyle habits or other health determinants were also included and categorised as cross-cutting. However, if the description of the interventions did not contain elements that could be traced back to potentially targeted risk factors, no effect was assumed on specific risk factors. Target age groups were defined based on the description of each intervention (i.e., childhood, adolescence, adulthood, old age, working age, women of childbearing age). The primary aim of all prevention interventions in RPPs is to reach the general population, although the interventions may vary in their directness. To provide a detailed view of the intervention nodes, we categorised the interventions based on whether the main target population was directly the (i) general population (e.g., providing walking groups and other free-access physical activity opportunities), or if the focus was on the involvement of (ii) policymakers (e.g., developing networks of active municipalities), (iii) health professionals (e.g., training health professionals on counselling and communication for the promotion of healthy lifestyles) or (iv) other stakeholders before reaching the general population.

### Checklist for the evaluation of RPPs

To describe the strengths and gaps of dementia prevention interventions in each region and autonomous province, we developed a bespoke quality checklist (Supplementary Table 2). Based on a toolkit for developing multisectoral action plans for non-communicable diseases published by the WHO, we structured our checklist according to the key elements of documents of interest to the prevention of dementia and the appraisal of prevention plans (5, 15, 16). The iterative process involved identifying and adapting the domain and items based on consensus, followed by a pilot phase of implementation of the checklist. The domain and items were identified to allow for a comprehensive characterisation of the elements considered necessary for designing, implementing, and monitoring public health interventions for the prevention of dementia. Afterwards, the research team agreed to keep only the most informative items referring to the content and characteristics of the RPPs. The checklist consists of the following four main domains and a total of 63 items:

- Domain 1, Demographic and epidemiological context of dementia (18 items);
- Domain 2, Burden of dementia (9 items);
- Domain 3, Prevention strategies for dementia (33 items);
- Domain 4, Feasibility of interventions (3 items).

Two researchers applied the checklist to each of the 21 RPPs, coding the presence or absence of each item (1 present – 0 absent). In case of disagreement, conflicts were resolved by involving a third researcher.

### Identification of non-dementia-specific preventive interventions

To provide a comprehensive overview of dementia prevention, we extended the search for preventive interventions beyond the MO1OS10, including interventions that were not explicitly described as targeting dementia but were, however, addressing the risk factors listed by the Lancet Commission. Specifically, we defined a search strategy to identify all interventions focusing on each of the 12 potentially modifiable risk factors for cognitive decline, according to the Lancet Commission (Supplemental Table 3) (5). The full text of all RPPs was analysed using the predefined search terms, and the identified interventions were collected and qualitatively described.

## Results

### Dementia-specific preventive interventions

**Figure 2:**
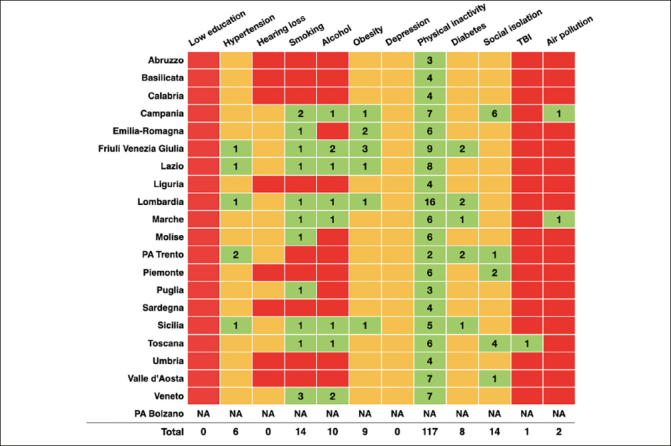
Dementia-specific (M01OS10) preventive interventions targeting potentially modifiable risk factors for cognitive decline at the subnational level in Italy Colour legend ∣ Green: risk factor directly addressed by an intervention; Orange: risk factor indirectly addressed by an intervention; Red: absence of interventions directly or indirectly addressing a risk factor. Numbers in green cells and “Total” row indicate how many times dementia-specific interventions addressed a specific risk factor. TBI: Traumatic Brain Injuries.

**Figure 3:**
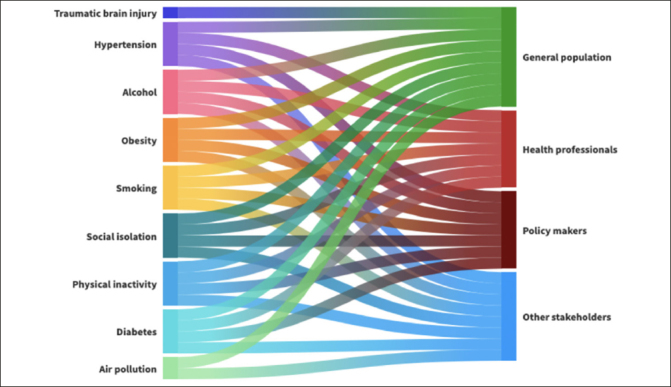
Main targets of the dementia-specific (M01OS10) preventive interventions by risk factor Graphic visualisation of who the main targets of the interventions were according to each risk factor.

Overall, the document analysis included 21 RPPs, 19 produced by regions and 2 by autonomous provinces. Only one RPP, issued by the autonomous province of Bolzano, was not organised into Macro and Specific Objectives as were the other RPPs and thus was excluded from the following analysis. When analysing the content of the 20 included RPPs, 21 Planned Programs and 9 Optional Programs were identified targeting MO1OS10, accounting for a total of 248 dementia preventive interventions (Supplementary Table 4). Out of 248 interventions, 122 targeted at least one of 12 risk factors identified by the Lancet Commission (5). All RPPs (20/20) defined PP02 «Active Communities» as a program where to include dementia preventive interventions, while one RPP (i.e., Lombardia) also defined PP03 «Health Promoting Workplaces» as a possible area for dementia prevention. In line with the PP02 «Active Communities», physical inactivity was the only risk factor included in all RPPs (20/20) as a direct target of 117/248 interventions for overall dementia prevention. Smoking was the target of dementia prevention strategies in more than half of the RPPs (11/20), with a total of 14 interventions. Less than half of the RPPs identified alcohol consumption (8/20) and obesity (6/20) as modifiable risk factors for dementia, with a total of 10 and 9 interventions, respectively. Only 25% of PRPs (5/20) included preventive interventions focusing on social isolation (n=14), hypertension (n=6), and diabetes (n=8). Two RPPs identified air pollution as a target of one of their preventive intervention, and only one RPP reported an intervention targeting head trauma. As reported in Figure 2, lower education, depression and hearing loss were not included as targets for dementia prevention in any of the analysed RPPs. Considering the interdependence among risk factors, all possible indirect effects of the interventions identified from the literature review are reported in orange in Figure 2. The analysis of direct effects and potential indirect effects of preventive interventions showed that the following three risk factors are covered in ≤10% of RPPS: education, head trauma, and air pollution. Moreover, 94 interventions did not address the Lancet Commission risk factors (e.g., “Breastfeeding and reading aloud for children’s health”) or did not address any risk factor (e.g., “Establishment of an inter-sectoral and multidisciplinary regional coordination table for the creation of a best practice model according to a ‘one health’ approach”). Lastly, 32 interventions were categorised as cross-cutting (e.g., “Promoting healthy lifestyles in specific contexts”). See Supplementary Table 4 for a detailed summary of the interventions and related risk factors. These interventions were not reported in Figure 2. When categorising the interventions according to the main target population, the general population resulted as the target of 97/248 (39%) preventive interventions, while health and social care professionals and policy-makers were the target of 63/248 (23%) and 81/248 (33%) interventions, respectively (Figure 3).

### Quality checklist application

**Figure 4:**
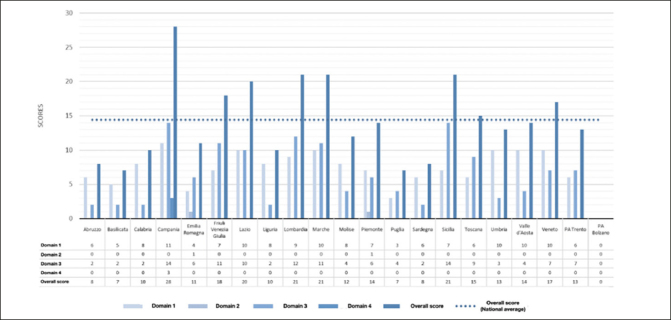
Dementia prevention bespoke quality checklist scores according to RPP Domain 1: Demographic and epidemiological context of dementia; Domain 2: Burden of dementia; Domain 3: Prevention strategies for dementia; Domain 4: Feasibility of interventions.

As reported in Figure 4, the total scores obtained from the RPPs were widely variable, ranging from a minimum score of 7/63 to a maximum score of 28/63. Due to the aforementioned limitations, we could not apply our checklist to the RPP of the autonomous province of Bolzano. When considering each domain of the checklist, Domain 1 - Demographic and epidemiological context of dementia - and Domain 3 - Prevention strategies for dementia - were the domains with the highest number of reported items. As for Domain 1, scores ranged from a minimum of 3 to a maximum of 11 out of 18 items. For a detailed report of scores for each domain for each regional plan, see Supplementary Table 5. Thanks to the operational surveillance systems in Italy (e.g., Passi and Passi d’Argento), data on the prevalence of different risk factors for cognitive decline were frequently available for Italian regions (17, 18). However, data on the overall prevalence of dementia were less frequent, and data on the specific prevalence of the two most common forms of dementia, Alzheimer’s dementia and vascular dementia, were even less frequent. The scores for Domain 3 ranged from a minimum of 2 to a maximum of 14 out of 33 items. Interventions targeting one single risk factor were the most frequent. For each intervention, we assessed the target age group. Due to how widely defined was the target population for each intervention, we observed that most interventions fit the age groups described by the Lancet Commission (5). RPPs rarely reported the references adopted for selecting preventive interventions for dementia and also rarely included interventions targeting individuals with cognitive impairment. None of the RPPs reported involving experts or establishing a dementia-specific working group. However, this lack may be due to the nature of the documents. Domain 2 - Dementia Burden - and Domain 4 - Feasibility of Interventions - were the domains for which the fewest number of items was reported. Specifically, items related to years of life lost (i.e., Emilia-Romagna) and years of life with disability (i.e., Piemonte) were reported only in the RPPs produced by these two regions. In both cases, data referred to all-cause dementia and no further details were provided on specific dementia subtypes. Only one region (i.e., Campania) reported the items included in Domain 4.

### Non-dementia-specific preventive interventions

As a further analysis, we assessed all interventions addressing specific risk factors for cognitive decline, as described in the Planned and Optional Programs of the RPPs, by applying a predefined set of keywords (see Methods and Supplementary Table 3). Despite observing a predictable increase in the number of addressed risk factors by each region, none of the RPPs simultaneously targeted all twelve risk factors. Moreover, almost one-third of the RPPs did not include interventions directly targeting depression and social isolation. Only one RPP (i.e., Lombardia) included a direct intervention targeting hearing loss.

## Discussion

### Strengths and gaps of population-level interventions for dementia prevention in Italy

Our document analysis of the RPPs for the years 2020–2025 aimed at describing all health policies targeted at dementia prevention in Italy. Previous studies emphasised the importance and potential outcomes of this analysis, but only one study specifically analysed primary prevention strategies at a sub-national level (19, 20). In line with Target 3.4 of the UN Agenda for Sustainable Development, Italy dedicated an entire Macro Objective (i.e., MO1) of the NPP 2020–2025 to reducing the potentially preventable burden of morbidity, mortality, and disability associated with non-communicable diseases (21). Dementia prevention was listed as one of the ten Specific Objectives of MO1, considering the significant social and economic costs associated with this condition (12). All RPPs identified physical inactivity as an area of intervention for dementia prevention. The most common framework adopted for this purpose was the Planned Program 02 (i.e., Active Communities). All interventions were in line with the life-course approach recommended by the WHO, ranging from school to old age (6, 7). Involving younger individuals is crucial to counteract the increasingly sedentary lifestyle trend observed in Italy in recent years (22). Moreover, involving older people answers the Lancet Commission’s indications that a sedentary lifestyle in this age group is responsible for 1.6% of the weighted population attributable fraction (PAF) of dementia (5). Assessing the proposed interventions targeting physical inactivity, we observed that social activities were the most prevalent category, with walking groups being the most common among them. These activities can also synergistically affect social isolation, which is another potentially modifiable risk factor for cognitive decline later in life. However, aside from the indirect effect of these interventions, we observed an overall lack of interventions specifically targeting social isolation in all RPPs. This is concerning, considering the latest findings from the Passi d’Argento surveillance system, which showed that almost 16% of individuals aged 65 and over in Italy have no social interactions during the week, and up to 75% do not participate in group activities (23). Considering that people aged 65 and older are currently 23.7% of the total population and are expected to increase by 13.4% within 2050, our future agendas should include late-life social isolation among their priorities (24). Overall, we observed that most of the RPPs included interventions targeting behavioural and intermediate cardiovascular risk factors other than physical inactivity (25). Most RPPs included smoking, excessive alcohol consumption, and obesity as common targets of interventions specifically aimed at dementia prevention. Moreover, when extending the analysis of RPPs beyond the Macro Objective dedicated to dementia (i.e., MO1OS10), we also identified several interventions targeted at diabetes and hypertension. None of the RPPs identified education, depression, and hearing loss as action areas for dementia prevention. Nonetheless, it is worth noting that Planned Program 01 focuses on «Health promoting schools» as defined by the WHO, which includes interventions for promoting health literacy and healthy lifestyles in school age (26). However, none of the RPPs included any such interventions in the context of dementia prevention. The NPP includes mental health as a topic of interest and considers it among NCDs. However, in the RPPs, depression was only indirectly addressed within interventions aimed at preventing dementia by targeting different risk factors. Very few unevenly distributed interventions specifically targeted depression, with none of them being included in the MO1OS10. We believe that the future agenda should focus on including a wider variety of interventions targeting this risk factor for two main reasons. As stated in the NPP, people living with depression can be included in different categories, such as brain health, mental health, and NCDs, as they are at higher risk of suffering from unhealthy lifestyles that can lead to different conditions (12). Moreover, according to the Lancet Commission, depression later in life is responsible for a weighted PAF of 3.9%, making it the fourth highest potentially modifiable risk factor linked to the greatest preventable burden (5). When considering hearing loss, we observed a significant gap in the primary prevention agenda for dementia prevention in Italy. Despite being the potentially modifiable risk factor with the highest PAF (i.e., 8.2%), only one intervention was identified specifically targeting it and was not included in the MO1OS10 (5). Hearing loss was only indirectly affected by interventions targeted to other risk factors or by health policies related to high-risk work environments. Our quality checklist showed that RPPs evenly characterised the distribution of potentially modifiable risk factors across regions, suggesting a widespread awareness of and access to the Passi and Passi d’Argento surveillance systems. However, data on the epidemiology and burden of dementia and the identification of specific targets for monitoring and assessing preventive interventions were the areas with the least amount of information. This finding is in line with previous research and hinders the estimation of the actual impact of preventive interventions on the distribution of risk factors and the prevalence of dementia (27). As evaluating the impact of strategies is essential for targeting future activities and funds based on an evidence-based approach, process and outcome indicators should be better defined.

To conclude, interventions targeting cardiovascular risk factors have the advantage of synergistically and simultaneously addressing several non-communicable diseases. However, the predominant number of interventions focusing on cardiovascular risk factors in a Specific Objective dedicated to dementia, such as the MO1OS10, may suggest that policymakers might not be fully aware of the existence and relevance of more specific risk factors for dementia (e.g., traumatic brain injury and hearing loss). This finding is supported by the lack of any reference to internationally validated documents and recommendations within the RPPs and is consistent with the knowledge profile of English policymakers described by Walsh et al (28).

### Limitations of the study

We acknowledge three main limitations in our study. In our document analysis of the Italian RPPs (2020–2025), our main goal was to provide a quantitative description of health policies addressing the risk factors for dementia, as defined by the Lancet Commission. Firstly, we acknowledge the limitation of only considering the binary variable of the presence or absence of interventions. However, we believe that this approach effectively fulfils the purpose of the present study by providing a clear and concise overview of the policy landscape. Nonetheless, in the future, implementation research frameworks and tools should be integrated to allow for more robust qualitative analyses of the health policy development process. A second limitation was defining the potential indirect effect of some interventions based on available evidence of a relationship between two factors rather than on the content of the preventive interventions themselves. This means we identified interventions that could have further effects in addition to the specific relationships identified. For example, encouraging the creation of groups to walk children to school could help reduce air pollution, and raising awareness of the risks of drunk driving could also help prevent traumatic brain injuries. Although considering only meta-analysis or systematic reviews can be seen as a strength in defining the potential indirect benefit of interventions, we acknowledge that other types of publications are available on this topic and will be the focus of future evaluation to ensure a more comprehensive and systematic evaluation of the plausibility of the interventions and their interconnectedness. The third element was the categorisation of interventions based on consensus, which does not allow the exclusion of a degree of subjectivity from the examiner, as the RPPs did not code the main target audience of interventions.

### Toward the implementation of the neurological quadrangle

To our knowledge, this study is the first attempt to evaluate the population-oriented intervention agenda for the prevention of dementia at the national and subnational levels. Our data highlight the existence of a know-do gap in including population approaches for the prevention of dementia in Italian health policies. The existing gap underscores the importance of involving policymakers, public health professionals, and dementia experts in co-designing population-level interventions. One potential solution to bridge the gap between data generation and data use is to employ logic models to facilitate such a participatory approach (29). In this approach, public health professionals would primarily be responsible for identifying evidence-based preventive interventions and selecting indicators (input, process, output, outcome, and impact). At the same time, dementia experts would assess the plausibility of the rationale and articulate the causal relationship between risk factors and cognitive decline (30, 31). This process will require an iterative collaboration and feedback from policymakers involved in the development of each RPP, who should be knowledgeable about the specific health profiles of the sub-national territory under their responsibility. Existing evidence-informed policy-making tools could facilitate this collaboration, hopefully leading to the institutionalisation of knowledge translation processes and ultimately preventing a significant number of dementia cases (32–34). Minimising the fragmentation and duplication of interventions while maximising their cost-effectiveness is a moral imperative as it helps reduce inequitable use of resources and disparities in population coverage (35, 36). Recognising the complex interconnectedness of dementia and NCDs, systems thinking is a valuable approach to planning prevention policies for these conditions and leveraging their commonalities (37). A key principle of systems thinking is to evaluate how the implementation of a new policy fits into an existing system. In Italy, adopting a systems lens would require the alignment, at both the national and sub-national levels, of the three main operational plans focusing on dementia and NCDs: the National Prevention Plan, the National Chronicity Plan, and the National Dementia Plan (12, 38, 39). In the future, the generalizability of our national experience should be tested in the context of a transnational, intersectoral, and multi-professional collaborative effort to implement the neurological quadrangle (i.e., the “Surveillance, research, and innovation” and “Prevention and promotion” pillars) (40). Such effort would substantially enhance the impact evaluation of universal dementia prevention, facilitating transparent knowledge sharing and supporting global programmes (41).

## Electronic supplementary material

Universal Prevention of Dementia in Italy: A Document Analysis of the 21 Italian Regional Prevention Plans, approximately 2.72 MB.

## Notes

*Ethical standards:* The article analyses data from publicly accessible documents and does not contain studies involving human participants or animals.
