# Supplementary material for: Universal Prevention of Dementia in Italy: A Document Analysis of the 21 Italian Regional Prevention Plans
Source: J Prev Alzheimers Dis. 2024 Jul 11;11(6):1525–33. doi: 10.14283/jpad.2024.144 (PMC11573845; doi:10.14283/jpad.2024.144)
Supplement: Supplementary file 1 — Universal Prevention of Dementia in Italy: A Document Analysis of the 21 Italian Regional Prevention Plans, approximately 2.72 MB. [file 42414_2024_144_MOESM1_ESM.docx]

**Universal prevention of dementia in Italy: A document analysis of the 21 Italian Regional Prevention Plans - SUPPLEMENTARY MATERIAL**

| **Contents** |  |
| --- | --- |
|  |  |
| **Table 1** - Structured review of plausible relationships between risk factors. | [Pg 2] |
| **Table 2** - Bespoke quality checklist. | [Pg 9] |
| **Table 3** - Keywords of the search strategy for non-dementia-specific interventions. | [Pg 10] |
| **Table 4** - Summary table of population-level interventions for dementia prevention divided by each RPP. | [Pg 11] |
| **Table 5** - Dementia prevention bespoke quality checklist disaggregated scores according to RPP. | [Pg 24] |
| **Table 6** - List of Planned Programs and Optional Programs addressing the 12 risk factors by RPP. | [Pg 25] |
| **Figure 1** - Planned Programs (PP) and Optional Programs (OP) including dementia-specific (M01OS10) preventive interventions by RPP. | [Pg 26] |
| **Figure 2** - Risk factors addressed by dementia-specific (M01OS10) preventive interventions by RPP. | [Pg 27] |

***Table 1*** *- Structured review of plausible relationships between risk factors.*

| **Risk factor targeted** | | **Risk factor possibly affected** |
| --- | --- | --- |
| **Hypertension** | | **Hearing Loss** |
| Li, Peishan, Kaiyun Pang, Rong Zhang, Lan Zhang, and Hui Xie. 2023. “Prevalence and Risk Factors of Hearing Loss among the Middle-Aged and Older Population in China: A Systematic Review and Meta-Analysis.” *European Archives of Oto-Rhino-Laryngology*. https://doi.org/10.1007/s00405-023-08109-3. | | |
| **Smoking** | | **Diabetes** |
| Larsson, Susanna C., and Stephen Burgess. 2022. “Appraising the Causal Role of Smoking in Multiple Diseases: A Systematic Review and Meta-Analysis of Mendelian Randomization Studies.” *EBioMedicine* 82. https://doi.org/10.1016/j.ebiom.2022.104154. | | |
| Pan, An, Yeli Wang, Mohammad Talaei, Frank B. Hu, and Tangchun Wu. 2015. “Relation of Active, Passive, and Quitting Smoking with Incident Type 2 Diabetes: A Systematic Review and Meta-Analysis.” *The Lancet Diabetes and Endocrinology* 3 (12): 958–67. https://doi.org/10.1016/S2213-8587(15)00316-2. | | |
| Akter, Shamima, Atsushi Goto, and Tetsuya Mizoue. 2017. “Smoking and the Risk of Type 2 Diabetes in Japan: A Systematic Review and Meta-Analysis.” Journal of Epidemiology. https://doi.org/10.1016/j.je.2016.12.017. | | |
| **Smoking** | | **Hearing Loss** |
| Li, Xiaowen, Xing Rong, Zhi Wang, and Aihua Lin. 2020. “Association between Smoking and Noise-Induced Hearing Loss: A Meta-Analysis of Observational Studies.” *International Journal of Environmental Research and Public Health*. https://doi.org/10.3390/ijerph17041201. | | |
| Nomura, Kyoko, Mutsuhiro Nakao, and Takeshi Morimoto. 2005. “Effect of Smoking on Hearing Loss: Quality Assessment and Meta-Analysis.” *Preventive Medicine* 40 (2): 138–44. https://doi.org/10.1016/j.ypmed.2004.05.011. | | |
| **Smoking** | | **Obesity** |
| Baalbaki, Reem, Leila Itani, Lara El Kebbi, Rawan Dehni, Nermine Abbas, Razan Farsakouri, Dana Awad, et al. 2019. “Association between Smoking Hookahs (Shishas) and Higher Risk of Obesity: A Systematic Review of Population-Based Studies.” *Journal of Cardiovascular Development and Disease*. https://doi.org/10.3390/jcdd6020023 | | |
| Park, Sehoon, Seong Geun Kim, Soojin Lee, Yaerim Kim, Semin Cho, Kwangsoo Kim, Yong Chul Kim, et al. 2023. “Causal Effects from Tobacco Smoking Initiation on Obesity-Related Traits: A Mendelian Randomization Study.” *International Journal of Obesity*. https://doi.org/10.1038/s41366-023-01371-9. | | |
| Lee, Won Jun, Ji Eun Lim, Ji One Kang, Tae Woong Ha, Hae Un Jung, Dong Jun Kim, Eun Ju Baek, Han Kyul Kim, Ju Yeon Chung, and Bermseok Oh. 2022. “Smoking-Interaction Loci Affect Obesity Traits: A Gene-Smoking Stratified Meta-Analysis of 545,131 Europeans.” *Lifestyle Genomics*. https://doi.org/10.1159/000525749. | | |
| **Alcohol** | | **Hypertension** |
| Liu, Feiyan, Yu Liu, Xizhuo Sun, Zhaoxia Yin, Honghui Li, Kunpeng Deng, Yang Zhao, et al. 2020. “Race- and Sex-Specific Association between Alcohol Consumption and Hypertension in 22 Cohort Studies: A Systematic Review and Meta-Analysis.” *Nutrition, Metabolism and Cardiovascular Diseases* 30 (8): 1249–59. https://doi.org/10.1016/j.numecd.2020.03.018. | | |
| Jung, Mi Hyang, Ein Soon Shin, Sang Hyun Ihm, Jin Gyu Jung, Hae Young Lee, and Cheol Ho Kim. 2020. “The Effect of Alcohol Dose on the Development of Hypertension in Asian and Western Men: Systematic Review and Meta-Analysis.” *Korean Journal of Internal Medicine* 35 (4): 906–16. https://doi.org/10.3904/KJIM.2019.016. | | |
| Zhong, Lixian, Weiwei Chen, Tonghua Wang, Qiuting Zeng, Leizhen Lai, Junlong Lai, Junqin Lin, and Shaohui Tang. 2022. “Alcohol and Health Outcomes: An Umbrella Review of Meta-Analyses Base on Prospective Cohort Studies.” *Frontiers in Public Health* 10. https://doi.org/10.3389/fpubh.2022.859947. | | |
| **Alcohol** | | **Smoking** |
| Amsterdam, Jan van, and Wim van den Brink. 2023. “The Effect of Alcohol Use on Smoking Cessation: A Systematic Review.” *Alcohol*. https://doi.org/10.1016/j.alcohol.2022.12.003. | | |
| **Alcohol** | | **Hearing Loss** |
| Qian, Peiyi, Zhixin Zhao, Shuangyan Liu, Jiarui Xin, Yun Liu, Yinzhu Hao, Yaxin Wang, and Lei Yang. 2023. “Alcohol as a Risk Factor for Hearing Loss: A Systematic Review and Meta-Analysis.” *PLoS ONE* 18 (1 January). https://doi.org/10.1371/journal.pone.0280641. | | |
| Lin, Rui Jun, Randall Krall, Brian D. Westerberg, Neil K. Chadha, and Justin K. Chau. 2012. “Systematic Review and Meta-Analysis of the Risk Factors for Sudden Sensorineural Hearing Loss in Adults.” *Laryngoscope*. https://doi.org/10.1002/lary.22480 | | |
| Chiao Amanda, Hughes ML, Karimuddanahalli Premkumar P, Zoucha K. 2024. “The Effects of Substance Misuse on Auditory and Vestibular Function: A Systematic Review.” *Ear Hear*. http://doi.org/10.1097/AUD.0000000000001425. | | |
| **Alcohol** | | **Obesity** |
| Siegmann, Eva Maria, Massimiliano Mazza, Christian Weinland, Falk Kiefer, Johannes Kornhuber, Christiane Mühle, and Bernd Lenz. 2022. “Meta-Analytic Evidence for a Sex-Diverging Association between Alcohol Use and Body Mass Index.” *Scientific Reports* 12 (1). https://doi.org/10.1038/s41598-022-25653-w. | | |
| Golzarand, Mahdieh, Asma Salari-Moghaddam, and Parvin Mirmiran. 2022. “Association between Alcohol Intake and Overweight and Obesity: A Systematic Review and Dose-Response Meta-Analysis of 127 Observational Studies.” *Critical Reviews in Food Science and Nutrition*. https://doi.org/10.1080/10408398.2021.1925221. | | |
| Llamosas-Falcón L, Rehm J, Bright S, Buckley C, Carr T, Kilian C, Lasserre AM, Lemp JM, Zhu Y, Probst C., 2023. “The Relationship Between Alcohol Consumption, BMI, and Type 2 Diabetes: A Systematic Review and Dose-Response Meta-analysis.” *Diabetes Care.* https://doi.org/ 10.2337/dc23-1015 | | |
| **Alcohol** | | **Depression** |
| Li, Jiande, Hongxuan Wang, Mei Li, Qingyu Shen, Xiangpen Li, Yuanpei Zhang, Jialing Peng, Xiaoming Rong, and Ying Peng. 2020. “Effect of Alcohol Use Disorders and Alcohol Intake on the Risk of Subsequent Depressive Symptoms: A Systematic Review and Meta-Analysis of Cohort Studies.” *Addiction*. https://doi.org/10.1111/add.14935. | | |
| Puddephatt, Jo Anne, Patricia Irizar, Andrew Jones, Suzanne H. Gage, and Laura Goodwin. 2022. “Associations of Common Mental Disorder with Alcohol Use in the Adult General Population: A Systematic Review and Meta-Analysis.” *Addiction*. https://doi.org/10.1111/add.15735. | | |
| Fredman Stein, Kim, Jennifer L. Allen, Ross Robinson, Cassandra Smith, Katherine Sawyer, and Gemma Taylor. 2022. “Do Interventions Principally Targeting Excessive Alcohol Use in Young People Improve Depression Symptoms?: A Systematic Review and Meta-Analysis.” *BMC Psychiatry* 22 (1). https://doi.org/10.1186/s12888-022-04006-x. | | |
| **Obesity** | | **Hypertension** |
| Natsis, Michail, Christina Antza, Ioannis Doundoulakis, Stella Stabouli, and Vasilios Kotsis. 2019. “Hypertension in Obesity: Novel Insights.” *Current Hypertension Reviews* 16 (1): 30–36. https://doi.org/10.2174/1573402115666190415154603. | | |
| Shariq, Omair A., and Travis J. Mckenzie. 2020. “Obesity-Related Hypertension: A Review of Pathophysiology, Management, and the Role of Metabolic Surgery.” *Gland Surgery*. https://doi.org/10.21037/gs.2019.12.03. | | |
| **Obesity** | | **Depression** |
| Sutaria, Shailen, Delan Devakumar, Sílvia Shikanai Yasuda, Shikta Das, and Sonia Saxena. 2019. “Is Obesity Associated with Depression in Children? Systematic Review and Meta-Analysis.” *Archives of Disease in Childhood* 104 (1): 64–74. https://doi.org/10.1136/archdischild-2017-314608. | | |
| Rao, Wen Wang, Qian Qian Zong, Ji Wen Zhang, Feng Rong An, Todd Jackson, Gabor S. Ungvari, Yifan Xiang, Ying Ying Su, Carl D’Arcy, and Yu Tao Xiang. 2020. “Obesity Increases the Risk of Depression in Children and Adolescents: Results from a Systematic Review and Meta-Analysis.” *Journal of Affective Disorders*. https://doi.org/10.1016/j.jad.2020.01.154. | | |
| Jokela, Markus, and Michael Laakasuo. 2023. “Obesity as a Causal Risk Factor for Depression: Systematic Review and Meta-Analysis of Mendelian Randomization Studies and Implications for Population Mental Health.” *Journal of Psychiatric Research* 163: 86–92. https://doi.org/10.1016/j.jpsychires.2023.05.034. | | |
| **Obesity** | | **Diabetes** |
| Riaz, Haris, Muhammad Shahzeb Khan, Tariq Jamal Siddiqi, Muhammad Shariq Usman, Nishant Shah, Amit Goyal, Sadiya S. Khan, Farouk Mookadam, Richard A. Krasuski, and Haitham Ahmed. 2018. “Association Between Obesity and Cardiovascular Outcomes: A Systematic Review and Meta-Analysis of Mendelian Randomization Studies.” *JAMA Network Open* 1 (7): e183788. https://doi.org/10.1001/jamanetworkopen.2018.3788. | | |
| **Diabetes** | | **Depression** |
| Hasan, Syed Shahzad, Abdullah A. Mamun, Alexandra M. Clavarino, and Therese Kairuz. 2015. “Incidence and Risk of Depression Associated with Diabetes in Adults: Evidence from Longitudinal Studies.” *Community Mental Health Journal* 51 (2): 204–10. https://doi.org/10.1007/s10597-014-9744-5. | | |
| Nouwen, A., K. Winkley, J. Twisk, C. E. Lloyd, M. Peyrot, K. Ismail, and F. Pouwer. 2010. “Type 2 Diabetes Mellitus as a Risk Factor for the Onset of Depression: A Systematic Review and Meta-Analysis.” *Diabetologia* 53 (12): 2480–86. https://doi.org/10.1007/s00125-010-1874-x. | | |
| Farooqi, Aaisha, Clare Gillies, Harini Sathanapally, Sophia Abner, Sam Seidu, Melanie J. Davies, William H. Polonsky, and Kamlesh Khunti. 2022. “A Systematic Review and Meta-Analysis to Compare the Prevalence of Depression between People with and without Type 1 and Type 2 Diabetes.” *Primary Care Diabetes*. https://doi.org/10.1016/j.pcd.2021.11.001. | | |
| **Diabetes** | | **Hearing Loss** |
| Akinpelu, Olubunmi V., Mario Mujica-Mota, and Sam J. Daniel. 2014. “Is Type 2 Diabetes Mellitus Associated with Alterations in Hearing? A Systematic Review and Meta-Analysis.” *Laryngoscope*. https://doi.org/10.1002/lary.24354. | | |
| Horikawa, Chika, Satoru Kodama, Shiro Tanaka, Kazuya Fujihara, Reiko Hirasawa, Yoko Yachi, Hitoshi Shimano, Nobuhiro Yamada, Kazumi Saito, and Hirohito Sone. 2013. “Diabetes and Risk of Hearing Impairment in Adults: A Meta-Analysis.” *Journal of Clinical Endocrinology and Metabolism* 98 (1): 51–58. https://doi.org/10.1210/jc.2012-2119. | | |
| **Traumatic Brain Injury** | | **Hearing Loss** |
| Chen, Jenny X., Michael Lindeborg, Seth D. Herman, Reuven Ishai, Renata M. Knoll, Aaron Remenschneider, David H. Jung, and Elliott D. Kozin. 2018. “Systematic Review of Hearing Loss after Traumatic Brain Injury without Associated Temporal Bone Fracture.” *American Journal of Otolaryngology - Head and Neck Medicine and Surgery*. https://doi.org/10.1016/j.amjoto.2018.01.018. | | |
| Šarkić, Bojana, Jacinta M. Douglas, and Andrea Simpson. 2019. “Peripheral Auditory Dysfunction Secondary to Traumatic Brain Injury: A Systematic Review of Literature.” *Brain Injury*. https://doi.org/10.1080/02699052.2018.1539868. | | |
| **Air pollution** | | **Hypertension** |
| Yang, Bo Yi, Zhengmin Qian, Steven W. Howard, Michael G. Vaughn, Shu Jun Fan, Kang Kang Liu, and Guang Hui Dong. 2018. “Global Association between Ambient Air Pollution and Blood Pressure: A Systematic Review and Meta-Analysis.” *Environmental Pollution*. https://doi.org/10.1016/j.envpol.2018.01.001. | | |
| Yan, Mengfan, Jiahui Xu, Chaokang Li, Pengyi Guo, Xueli Yang, and Nai jun Tang. 2021. “Associations between Ambient Air Pollutants and Blood Pressure among Children and Adolescents: A Systemic Review and Meta-Analysis.” *Science of the Total Environment*. https://doi.org/10.1016/j.scitotenv.2021.147279. | | |
| Qin, Pei, Xinping Luo, Yunhong Zeng, Yanyan Zhang, Yang Li, Yuying Wu, Minghui Han, et al. 2021. “Long-Term Association of Ambient Air Pollution and Hypertension in Adults and in Children: A Systematic Review and Meta-Analysis.” *Science of the Total Environment*. https://doi.org/10.1016/j.scitotenv.2021.148620. | | |
| **Air pollution** | | **Physical Inactivity** |
| An, Ruopeng, Jing Shen, Binbin Ying, M. Tainio, Zorana Jovanovic Andersen, and Audrey de Nazelle. 2019. “Impact of Ambient Air Pollution on Physical Activity and Sedentary Behavior in China: A Systematic Review.” *Environmental Research*. https://doi.org/10.1016/j.envres.2019.108545. | | |
| An, Ruopeng, Sheng Zhang, Mengmeng Ji, and Chenghua Guan. 2018. “Impact of Ambient Air Pollution on Physical Activity among Adults: A Systematic Review and Meta-Analysis.” *Perspectives in Public Health*. https://doi.org/10.1177/1757913917726567. | | |
| **Air pollution** | | **Diabetes** |
| Aarthi, Garudam Raveendiran, Thaharullah Shah Mehreen Begum, Suzana Al Moosawi, Dian Kusuma, Harish Ranjani, Rajendra Paradeepa, Venkatasubramanian Padma, Viswanathan Mohan, Ranjit Mohan Anjana, and Daniela Fecht. 2023. “Associations of the Built Environment with Type 2 Diabetes in Asia: A Systematic Review.” *BMJ Open*. https://doi.org/10.1136/bmjopen-2022-065431. | | |
| Balti, Eric V., Justin B. Echouffo-Tcheugui, Yandiswa Y. Yako, and Andre P. Kengne. 2014. “Air Pollution and Risk of Type 2 Diabetes Mellitus: A Systematic Review and Meta-Analysis.” *Diabetes Research and Clinical Practice* 106 (2): 161–72. https://doi.org/10.1016/j.diabres.2014.08.010. | | |
| Liu, Feifei, Gongbo Chen, Wenqian Huo, Chongjian Wang, Suyang Liu, Na Li, Shuyuan Mao, Yitan Hou, Yuanan Lu, and Hao Xiang. 2019. “Associations between Long-Term Exposure to Ambient Air Pollution and Risk of Type 2 Diabetes Mellitus: A Systematic Review and Meta-Analysis.” *Environmental Pollution*. https://doi.org/10.1016/j.envpol.2019.06.033. | | |
| **Air pollution** | | **Depression** |
| Zeng, Ying, Ruoheng Lin, Li Liu, Yiting Liu, and Yamin Li. 2019. “Ambient Air Pollution Exposure and Risk of Depression: A Systematic Review and Meta-Analysis of Observational Studies.” *Psychiatry Research*. https://doi.org/10.1016/j.psychres.2019.04.019. | | |
| Fan, Shu Jun, Joachim Heinrich, Michael S. Bloom, Tian Yu Zhao, Tong Xing Shi, Wen Ru Feng, Yi Sun, et al. 2020. “Ambient Air Pollution and Depression: A Systematic Review with Meta-Analysis up to 2019.” *Science of the Total Environment*. https://doi.org/10.1016/j.scitotenv.2019.134721. | | |
| Borroni, Elisa, Angela Cecilia Pesatori, Valentina Bollati, Massimiliano Buoli, and Michele Carugno. 2022. “Air Pollution Exposure and Depression: A Comprehensive Updated Systematic Review and Meta-Analysis.” *Environmental Pollution*. https://doi.org/10.1016/j.envpol.2021.118245 | | |
| **Air pollution** | | **Obesity** |
| Malacarne, Diego, Evangelos Handakas, Oliver Robinson, Elisa Pineda, Marc Saez, Leda Chatzi, and Daniela Fecht. 2022. “The Built Environment as Determinant of Childhood Obesity: A Systematic Literature Review.” *Obesity Reviews*. https://doi.org/10.1111/obr.13385. | | |
| Lin, Lisen, Tianyu Li, Mengqi Sun, Qingqing Liang, Yuexiao Ma, Fenghong Wang, Junchao Duan, and Zhiwei Sun. 2022. “Global Association between Atmospheric Particulate Matter and Obesity: A Systematic Review and Meta-Analysis.” *Environmental Research*. https://doi.org/10.1016/j.envres.2022.112785 | | |
| Zheng, Jingying, Huiling Zhang, Jianyang Shi, Xin Li, Jing Zhang, Kunlun Zhang, Yameng Gao, Jingtong He, Jianghong Dai, and Juan Wang. 2024. “Association of Air Pollution Exposure with Overweight or Obesity in Children and Adolescents: A Systematic Review and Meta–Analysis.” *Science of The Total Environment* 910 (February): 168589. https://doi.org/10.1016/J.SCITOTENV.2023.168589. | | |
| **Social Isolation** | | **Depression** |
| Erzen, Evren, and Özkan Çikrikci. 2018. “The Effect of Loneliness on Depression: A Meta-Analysis.” *International Journal of Social Psychiatry*. https://doi.org/10.1177/0020764018776349. | | |
| Lambert Van As, Barbara Adriana, Enrico Imbimbo, Angela Franceschi, Ersilia Menesini, and Annalaura Nocentini. 2022. “The Longitudinal Association between Loneliness and Depressive Symptoms in the Elderly: A Systematic Review.” *International Psychogeriatrics*. https://doi.org/10.1017/S1041610221000399. | | |
| Almeida, Isabelle Lina de Laia, Jaqueline Ferraz Rego, Amanda Carvalho Girardi Teixeira, and Marília Rodrigues Moreira. 2021. “Social Isolation and Its Impact on Child and Adolescent Development: A Systematic Review.” *Revista Paulista de Pediatria : Orgao Oficial Da Sociedade de Pediatria de Sao Paulo*. https://doi.org/10.1590/1984-0462/2022/40/2020385. | | |
| **Physical Inactivity** | | **Obesity** |
| Silveira, Erika Aparecida, Carolina Rodrigues Mendonça, Felipe Mendes Delpino, Guilherme Vinícius Elias Souza, Lorena Pereira de Souza Rosa, Cesar de Oliveira, and Matias Noll. 2022. “Sedentary Behavior, Physical Inactivity, Abdominal Obesity and Obesity in Adults and Older Adults: A Systematic Review and Meta-Analysis.” *Clinical Nutrition ESPEN* 50: 63–73. https://doi.org/10.1016/j.clnesp.2022.06.001. | | |
| Cleven, Laura, Janina Krell-Roesch, Claudio R. Nigg, and Alexander Woll. 2020. “The Association between Physical Activity with Incident Obesity, Coronary Heart Disease, Diabetes and Hypertension in Adults: A Systematic Review of Longitudinal Studies Published after 2012.” *BMC Public Health* 20 (1). https://doi.org/10.1186/s12889-020-08715-4. | | |
| Miller, Clint T., Steve F. Fraser, Itamar Levinger, Nora E. Straznicky, John B. Dixon, John Reynolds, and Steve E. Selig. 2013. “The Effects of Exercise Training in Addition to Energy Restriction on Functional Capacities and Body Composition in Obese Adults during Weight Loss: A Systematic Review.” *PLoS ONE* 8 (11). https://doi.org/10.1371/journal.pone.0081692. | | |
| **Physical Inactivity** | | **Diabetes** |
| Aune, Dagfinn, Teresa Norat, Michael Leitzmann, Serena Tonstad, and Lars Johan Vatten. 2015. “Physical Activity and the Risk of Type 2 Diabetes: A Systematic Review and Dose-Response Meta-Analysis.” European Journal of Epidemiology. https://doi.org/10.1007/s10654-015-0056-z. | | |
| Cleven, Laura, Janina Krell-Roesch, Claudio R. Nigg, and Alexander Woll. 2020. “The Association between Physical Activity with Incident Obesity, Coronary Heart Disease, Diabetes and Hypertension in Adults: A Systematic Review of Longitudinal Studies Published after 2012.” *BMC Public Health* 20 (1). | | |
| Jayedi Ahmad, Zargar MS, Emadi A, Aune D. Walking speed and the risk of type 2 diabetes: a systematic review and meta-analysis.2023. *British Journal of Sports Medicine*. https://doi.org/10.1136/bjsports-2023-107336. | | |
| **Physical Inactivity** | | **Depression** |
| Pearce, Matthew, Leandro Garcia, Ali Abbas, Tessa Strain, Felipe Barreto Schuch, Rajna Golubic, Paul Kelly, et al. 2022. “Association between Physical Activity and Risk of Depression: A Systematic Review and Meta-Analysis.” JAMA Psychiatry. https://doi.org/10.1001/jamapsychiatry.2022.0609. | | |
| Schuch, Felipe B., Davy Vancampfort, Joseph Firth, Simon Rosenbaum, Philip B. Ward, Edson S. Silva, Mats Hallgren, et al. 2018. “Physical Activity and Incident Depression: A Meta-Analysis of Prospective Cohort Studies.” American Journal of Psychiatry. https://doi.org/10.1176/appi.ajp.2018.17111194. | | |
| Mammen, George, and Guy Faulkner. 2013. “Physical Activity and the Prevention of Depression: A Systematic Review of Prospective Studies.” *American Journal of Preventive Medicine*. https://doi.org/10.1016/j.amepre.2013.08.001. | | |
| **Physical Inactivity** | | **Social isolation** |
| Pels, Fabian, and Jens Kleinert. 2016. “Loneliness and Physical Activity: A Systematic Review.” International Review of Sport and Exercise Psychology. https://doi.org/10.1080/1750984X.2016.1177849. | | |
| **Physical Inactivity** | **Hypertension** | |
| Pescatello, Linda S., David M. Buchner, John M. Jakicic, Kenneth E. Powell, William E. Kraus, Bonny Bloodgood, Wayne W. Campbell, et al. 2019. “Physical Activity to Prevent and Treat Hypertension: A Systematic Review.” Medicine and Science in Sports and Exercise. | | |
| Liu, Xuejiao, Dongdong Zhang, Yu Liu, Xizhuo Sun, Chengyi Han, Bingyuan Wang, Yongcheng Ren, et al. 2017. “Dose-Response Association between Physical Activity and Incident Hypertension: A Systematic Review and Meta-Analysis of Cohort Studies.” *Hypertension*. https://doi.org/10.1161/HYPERTENSIONAHA.116.08994. | | |
| Edwards, Jamie J., Algis H.P. Deenmamode, Megan Griffiths, Oliver Arnold, Nicola J. Cooper, Jonathan D. Wiles, and Jamie M. O’Driscoll. 2023. “Exercise Training and Resting Blood Pressure: A Large-Scale Pairwise and Network Meta-Analysis of Randomised Controlled Trials.” *British Journal of Sports Medicine*. https://doi.org/10.1136/bjsports-2022-106503. | | |

***Table 2*** *- Bespoke quality checklist*


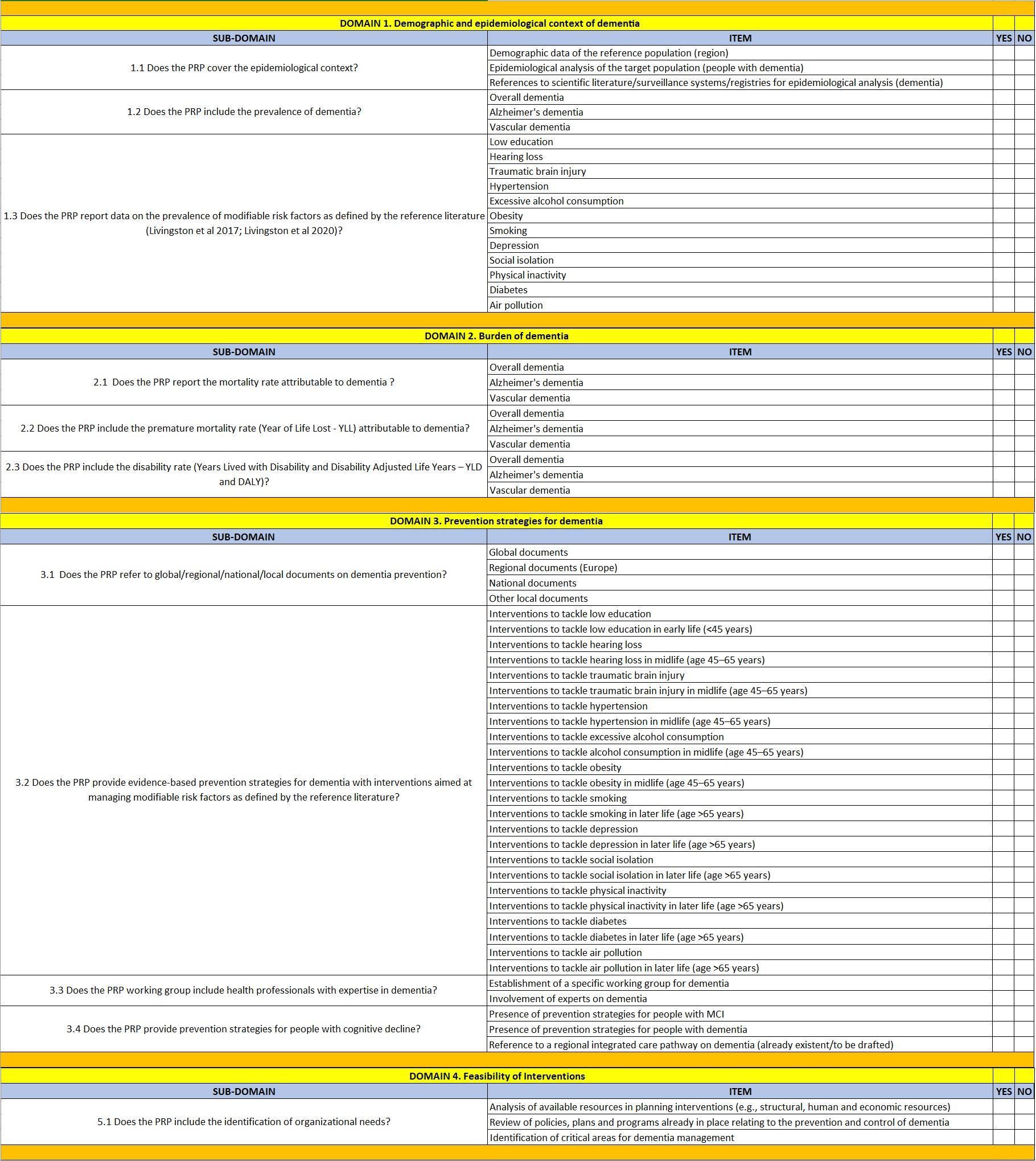


***Table 3*** *- Key words of the search strategy for non-dementia-specific interventions*

| **Ipertensione** |
| --- |
| iperte* – “pressione arteriosa” – “sale” |
| **Ipoacusia** |
| ipoacu* – sordità – “udito” – acustic* |
| **Obesità** |
| obes* – sovrappes* |
| **Depressione** |
| depress* – “umore” |
| **Diabete** |
| diabet* |
| **Inattività fisica** |
| fisic* – sport* – sedentar* – “inattività” – motor* |
| **Fumo** |
| fum* – “tabacco” – “nicotina” – tabagi* – sigarett* |
| **Alcol** |
| alcool* – alcol* |
| **Isolamento sociale** |
| “isolamento” – social* |
| **Traumi cranici** |
| commozione – cranic* – testa – stradal* |
| **Inquinamento atmosferico** |
| atmosferic* – “aria” – *door – “emissioni” |
| **Bassa scolarità** |
| scuol* – scola* – educa* – istru* – format* – formaz* |

***Table 4*** *- Summary table of population-level interventions for dementia prevention divided by each RPP*

| **RPP** | **PP/OP** | **Intervention** | **Risk factor** | **Main target** | **Life cycle** |
| --- | --- | --- | --- | --- | --- |
| **EMILIA-**  **ROMAGNA** | **PP02** | Gymnasium Network for Health and Sports Network for Health | physical inactivity | policy makers; other | childhood, adolescence, adulthood, old age |
|  |  | *Advocacy* for public spaces that promote movement and health | physical inactivity | policy maker | childhood, adolescence, adulthood, old age |
|  |  | Walking groups and other free-access motor activity opportunities | physical inactivity | general population | adulthood, old age |
|  |  | Promotion of sporting activity for people with disabilities and mentally ill people | physical inactivity | health professionals; policy makers; other | adulthood, old age |
|  |  | Health Map | physical inactivity, smoking | general population; health professionals | childhood, adolescence, adulthood, old age |
|  |  | Offering specific *counselling* on physical activity to people with chronic diseases | physical inactivity | general population | adulthood, old age |
|  | **OP20** | Connecting *counselling* provision and caretaking pathways with second-level health and non-health opportunities̀. | - | policy makers; other | - |
|  |  | Social marketing campaign on successful ageing | cross-cutting | general population | adulthood, old age |
|  |  | Caring for people with health risk behaviour | - | general population | - |
|  |  | Implementation of the regional model of organising a transversal preventive and clinical nutrition network in the AUSLs and implementation of PPDTAs for overweight and obese adults | obesity | policy makers; health professionals | childhood, adolescence, adulthood, old age |
|  |  | Short notice on health risk behaviour | - | healthcare professionals | - |
|  |  | PPDTA child with overweight and obesitỳ: data collection and evaluation by second-level multidisciplinary teams | obesity | other | childhood, adolescence |
| **ABRUZZO** | **PP02** | To renew existing agreements or create new ones with ANCI, universities, regional school office, CONI etc. To collaborate in the creation and dissemination of active communities. | - | policy maker | - |
|  |  | Exercise and sports activities among people with physical, psychic, sensory and/or mixed disabilities | physical inactivity | general population; health professionals | adulthood |
|  |  | Implementation of AFA, EFA programme | physical inactivity | general population; health professionals; other | adolescence, adulthood, old age |
|  |  | Realisation of a short motivational *counselling* training module | - | healthcare professionals | - |
|  |  | Adhesion of the municipality to the network of municipalities on the move | - | policy maker | - |
|  |  | Develop communication actions to make the population aware of the importance of movement and the opportunities that exist | physical inactivity | general population | adolescence, adulthood, old age |
|  |  | Physical activity and gender difference | - | general population | - |
| **BASILICATA** | **PP02** | Training of health professionals in *counselling* and communication for the promotion of healthy lifestyles | physical inactivity | health professionals, other | childhood, adolescence, adulthood, old age |
|  |  | Cross-sectoral interventions to promote an active lifestyle from an early age | physical inactivity | general population | childhood, adolescence, adulthood, old age |
|  |  | Colours of the seasons - programme in cooperation with the environment department | physical inactivity | general population | childhood, adolescence, adulthood, old age |
|  |  | Establishment of the Lucania network for the promotion of physical activities. | - | policy maker | - |
|  |  | Promotion of a regional law on active ageing | physical inactivity | general population | old age |
| **LIGURIA** | **PP02** | Census of municipal urban areas to be used for motor activities for citizens. | - | policy maker | - |
|  |  | Inclusion of curricular physical activity students with disabilities. | physical inactivity | general population | childhood, adolescence |
|  |  | Orientation to school-age sports practice | physical inactivity | general population | childhood, adolescence |
|  |  | Activation of Adapted Physical Activity Projects | physical inactivity | general population | adulthood, old age |
|  |  | Communities on the move | physical inactivity | general population | childhood, adolescence, adulthood, old age |
| **SARDEGNA** | **PP02** | Cross-cutting action: communication tools to increase citizens' ability to make healthy choices | physical inactivity | general population | childhood, adolescence, adulthood, old age |
|  |  | Transversal action: activation of training paths - joint action between stakeholders, and improvement of skills on motivational *counselling* in SSR operators, as recommended by the national chronicity plan | physical inactivity | healthcare professionals | adolescence, adulthood, old age |
|  |  | Promotion of physical activitỳ in young people, the adult population and the elderly to promote active and healthy ageing and social inclusion | physical inactivity | general population | childhood, adolescence, adulthood, old age |
|  |  | Cross-sectoral action: Consolidation of inter-sectoral and inter-institutional alliances for the promotion of healthy and active lifestyles in the community | - | policy maker | - |
|  |  | Promotion/prescription of physical activity and prescription of exercise in persons with intermediate risk factors or established chronic diseases, with particular attention to the most vulnerable and at increased risk of social exclusion | physical inactivity | general population | childhood, adolescence, adulthood, old age |
| **MOLISE** | **PP02** | Joint training healthcare-professional andtraining other sectors professionals | - | other | - |
|  |  | Creation of 'health maps' (opportunities for physical activity and active mobility) | physical inactivity | general population | childhood, adolescence, adulthood, old age |
|  |  | Realisation of pathways/programmes for the promotion of physical activity. | physical inactivity | general population | childhood, adolescence, adulthood, old age |
|  |  | Stakeholder involvement | - | other | - |
|  |  | 'Informa' communication campaign for the promotion of physical activity and healthy lifestyles | cross-cutting | general population | childhood, adolescence, adulthood, old age |
|  |  | Involvement of local authorities | physical inactivity | policy maker | childhood, adolescence, adulthood, old age |
|  |  | Brief *counselling* for change | - | healthcare professionals |  |
|  |  | Strengthening the alliance between schools and healthcare | physical inactivity, smoking | policy maker | childhood, adolescence |
|  |  | I am not fragile | physical inactivity | general population | adulthood, old age |
|  |  | Design and implementation of a population stratification programme in accordance with clinical risks and health and social needs | - | other | - |
|  |  | Health gyms: promoting physical exercise through the creation of a network of territorial gyms | physical inactivity | general population; health professionals; other | childhood, adolescence, adulthood, old age |
| **PUGLIA** | **PP02** | Strategies for reducing elderly people at risk of frailty | cross-cutting | general population | adulthood; old age |
|  |  | Identification of individuals at risk and/or suffering from MCNT | smoke | general population | childhood; adolescence; adulthood; working age; women of childbearing age |
|  |  | Living in the parks'- puglia.m.i.c.a. (integrated motor activity culture and environment) | physical inactivity | general population | adulthood; old age |
|  |  | Guidelines on motor activity for local authorities | physical inactivity | policy maker | childhood; adolescence; adulthood; working age; women of childbearing age |
|  |  | Offering pathways aimed at motor literacy | physical inactivity | general population | childhood; adolescence |
| **VALLE D'AOSTA** | **PP02** | Agreements to promote movement and physical activity with institutions | physical inactivity | policy maker | childhood, adolescence, adulthood, old age |
|  |  | Learning to promote a healthy and active lifestyle | - | healthcare professionals | - |
|  |  | *Health* and *health* literacy | - | healthcare professionals | - |
|  |  | Agreements to promote movement with sports and voluntary associations | physical inactivity | other | adolescence, adulthood, old age |
|  |  | Urban gardens in the municipality of Aosta | - | general population | - |
|  |  | Health-promoting gyms | physical inactivity | general population | childhood, adolescence, adulthood, old age |
|  |  | Agreements with old sector entities for the implementation of the PRP community programme | physical inactivity | other | childhood, adolescence, adulthood, old age |
|  |  | Mapping motor and sports activities in the region | physical inactivity | policy maker | childhood, adolescence, adulthood, old age |
|  |  | Brief counselling in health care | - | healthcare professionals | - |
|  |  | Breastfeeding and reading aloud for children's health | - | general population | - |
|  |  | Agreements with institutions to implement the RPP community programme | physical inactivity | policy maker | childhood, adolescence, adulthood, old age |
|  |  | Enhancing and communicating a natural territory that facilitates movement and healthy lifestyles | - | general population | - |
|  |  | Walking groups in the Aosta Valley | physical inactivity, social isolation | general population | adolescence, adulthood, old age |
| **LOMBARDIA** | **PP02** | Development of a social marketing strategy and communication tools for specific targets | physical inactivity | policy-maker | childhood; adolescence; adulthood; working age; women of childbearing age |
|  |  | Implementation of the regional training programme with pathways/provision aimed at specific targets on topics of interest | cross-cutting | health professionals; other | childhood; adolescence; adulthood; working age; women of childbearing age |
|  |  | Implementation of regional guidelines for the integration of BP for the promotion of physical activity and exercise, cross-sectoral and interdisciplinary intervention models, centred on *evidence-based* methodologies | physical inactivity | policy makers; other | working age |
|  |  | Inclusion in the annual preventive planning of the SSR (rules, addresses and objectives to the DGs ATS/ASST, ATS local integrated health promotion plans, etc.) of the offer of structured physical exercise or adapted physical activity (AFA) programmes aimed at the population of all age groups with one or more risk factors, specific pathologies or fragile conditions | physical inactivity | policy-maker | childhood; adolescence; adulthood; working age; women of childbearing age |
|  |  | Establishment of a permanent regional laboratory for the promotion of physical activity and exercise | physical inactivity | policy maker | childhood; adolescence; adulthood; working age; women of childbearing age |
|  |  | Increasing the supply of physical activity and exercise in the annual preventive planning of the SSR (rules, guidelines and objectives to the DGs ATS/ASST, ATS local integrated health promotion plans etc.) | physical inactivity | general population | childhood; adolescence; adulthood; working age; women of childbearing age |
|  |  | Implementation of the WHP model - health promoting workplaces - with BP for the promotion of physical activity/movement in the framework of *health ageing* | physical inactivity | policy-maker | working age |
|  |  | Drafting and dissemination of indications for the adoption of BPs provided for in the regional guidelines for the promotion of "active communities" within social planning (area plans) and the planning of the SSR (rules, guidelines and objectives to the DGs ATS/ASST, ATS local integrated health promotion plans etc.). | - | policy makers; other | - |
|  |  | Establishment of permanent local laboratories (ATS) for the promotion of awareness-raising activities and the provision of age-adapted physical and/or sports activity programmes | physical inactivity | general population; other | childhood; adolescence; adulthood; working age; women of childbearing age |
|  |  | Promotion of opportunities and provision of physical activity and exercise for the disabled through regional guidelines | physical inactivity | general population | childhood; adolescence; adulthood; working age; women of childbearing age |
|  |  | Implementation of regional training programme aimed at promoting strategies for physical activity and exercise with modules dedicated to: GPs, PLS, family and community nurses, management training, local police, planning offices | physical inactivity | health professionals; other | childhood; adolescence; adulthood; working age; women of childbearing age |
|  |  | Reducing the impact of risk factors for active ageing with a reduced burden of disease and disability through the implementation of regional guidelines for increasing the offer of programmes/pathways integrated in the annual planning of the SSR | cross-cutting | general population | childhood; adolescence; adulthood; working age; women of childbearing age |
|  |  | Awareness-raising and activation of local administrators for the promotion of physical activity and exercise in the framework of *urban health* through cross-sectoral agreements | physical inactivity | policy maker | childhood; adolescence; adulthood; working age; women of childbearing age |
|  |  | Promotion of physical activity and exercise within the regional planning of youth centres through regional best practice guidelines | physical inactivity | general population | adolescence |
|  |  | Implementation of the regional training programme to support the skills of all categories of professionals working for the promotion of health-enhancing lifestyles | cross-cutting | health professionals; other | working age |
|  |  | Implementation of the 'active communities' model at territorial level, with the activation of local workshops (involving the various stakeholders, universities, local authorities, ASST, sports movements and associations, sports facility managers, etc.). | physical inactivity | general population; other | childhood; adolescence; adulthood; working age; women of childbearing age |
|  |  | Development of regional guidelines for the definition and promotion of 'active communities'. | - | policy-maker | - |
|  |  | Implementation of the regional training programme based on the outcomes of the CCM project - central actions "sustaining change: distance learning to support the skills of professionals working for the promotion of health-promoting lifestyles", aimed at GPs, ATS/ASST personnel, family and community nurses, municipal social service operators | cross-cutting | health professionals; other | childhood; adolescence; adulthood; working age; women of childbearing age |
|  |  | Realisation with the involvement of the permanent regional laboratory for the promotion of physical activity and exercise: of 2.0 tools (e.g. "health offer around you" maps usable from *devices*) to facilitate health choices by the population; integration of information content in the "cancer screening" communication set | - | general population | - |
|  |  | Raising public awareness of the health benefits of physical activity and exercise by formalising regional agreements with strategic stakeholders | physical inactivity | policy-maker; other | childhood; adolescence; adulthood; working age; women of childbearing age |
|  |  | Implementation of regional training programme in cooperation with other regional DDGG for *caregivers* and carers | cross-cutting | health professionals; other | old age; working age |
|  |  | Promotion of physical activity and exercise in cooperation with SPS network Lombardy | physical inactivity | general population | childhood; adolescence; adulthood; |
|  |  | Establishment of local workshops in each ats and in each district (ATS/ASST) | - | general population; other |  |
|  |  | Increasing the level of physical activity in different age groups, facilitating the inclusion of socio-economically disadvantaged and frail individuals | physical inactivity | general population | childhood; adolescence; adulthood; working age; women of childbearing age |
|  | **PP03** | Implementation of targeted campaigns in cooperation with scientific societies | cross-cutting | general population | working age |
|  |  | Formalisation of a memorandum of understanding and cooperation with ANCI for the implementation of the WHP programme - local authorities | - | policy maker | - |
|  |  | Implementation of the motivational counselling offer according to the TTM model (*transtheoretical model of change*) | cross-cutting | healthcare professionals | working age |
|  |  | Formalisation of the memorandum of understanding and cooperation with the Ministry of Economy and Finance (MEF) for the implementation of the WHP - PA programme | cross-cutting | policy maker | working age |
|  |  | Implementation of the 'regional document of recommended and sustainable practices'. | - | policy makers; other | - |
|  |  | Systematic survey and evaluation of preventive and training programmes implemented in the workplace *setting* | cross-cutting | policy-maker | working age |
|  |  | Establishment of the regional inter-sectoral laboratory 'social marketing and communication for health in the workplace'. | cross-cutting | policy-maker; other | working age |
|  |  | Implementation of targeted campaigns in cooperation with employer/trade associations and trade unions and the consular corps | cross-cutting | policy-maker; other | working age |
|  |  | Formalisation of the document 'Recommended and sustainable practices in the WHP workplace - Lombardy'. | physical inactivity, smoking, alcohol | policy makers; other | working age |
|  |  | Formalisation of partnerships between the Lombardy region and system stakeholders for the dissemination of the health-promoting workplace model - WHP | cross-cutting | policy maker | working age |
|  |  | Definition of a system for the systematic collection and evaluation of the outcomes of the regional document of recommended practices | cross-cutting | policy-maker | working age |
|  |  | Promotion of the WHP Lombardy model and of adhesion to the network by private and public companies, within the annual preventive planning of the SSR | - | policy makers; other | - |
|  |  | Strengthening the impact of the Lombardy WHP model in workplaces with a low-skilled manual labour force by means of targeted regional guidelines and the inclusion of targeted pathways/modules in regional training programmes | - | health professionals; other | - |
|  | **OP20** | Definition of integrated preventive PDTA for the treatment of major chronic diseases | cross-cutting | policy-maker | childhood; adolescence; adulthood; working age; women of childbearing age |
|  |  | Implementation of operational tools and devices outcome of the 2019 CCM project "foodia-net" for patients with type 1 diabetes and mental health service patients | - | policy-makers; healthcare professionals | childhood; adolescence; adulthood; working age; women of childbearing age |
|  |  | Activation of structured partnerships with patient associations and drafting of guidelines at regional level | cross-cutting | other | childhood; adolescence; adulthood; working age; women of childbearing age |
|  |  | Definition and inclusion of topics within the regional training programme | cross-cutting | health professionals; other | childhood; adolescence; adulthood; working age; women of childbearing age |
|  |  | Targeted training offer on topics of interest within the regional training programme | cross-cutting | health professionals; other | childhood; adolescence; adulthood; working age; women of childbearing age |
|  |  | Definition of a targeted regional communication plan | cross-cutting | general population | childhood; adolescence; adulthood; working age; women of childbearing age |
|  |  | Implementation and systemisation of the sub-stratification of level 3 and 4 subjects (dgr 6164) in relation to the presence of intermediate risk factors | hypertension, diabetes, obesity | policy-makers; healthcare professionals | childhood; adolescence; adulthood; working age; women of childbearing age |
|  |  | Formalisation of regional alliances and partnerships (territorially declined) with sector stakeholders | - | policy makers; other | childhood; adolescence; adulthood; working age; women of childbearing age |
|  |  | Promoting scientific collaboration agreements and partnerships | cross-cutting | policy makers; other | childhood; adolescence; adulthood; working age; women of childbearing age |
|  |  | Improving the quality of care for specific types of chronic patients through the dissemination and implementation of the outcomes of the CCM project 2019 central actions in support of the Ministry of Health | diabetes | health professionals; other | childhood; adolescence; adulthood; working age; women of childbearing age |
| **SICILIA** | **PP02** | Prepare the regional policy document for the promotion of physical activity and reduction of sedentary behaviour, in line with the Global Action Plan for Physical Activity 2018-2030 | physical inactivity | general population | childhood; adolescence; adulthood; working age; women of childbearing age |
|  |  | "A mile a day around the school" | physical inactivity | general population | childhood |
|  |  | Good practice project on *the* promotion of physical activity in people over 65 | physical inactivity | general population | old age |
|  |  | Encouraging the promotion of physical activity in all age groups in safe, inclusive and easily accessible green areas and public spaces | physical inactivity | general population | adolescence; adulthood; working age; women of childbearing age |
|  | **OP11** | Integrated therapeutic care prevention pathway for the early identification of cognitive decline and dementia | physical inactivity, alcohol, smoking, hypertension, diabetes, obesity | general population | adulthood |
|  |  | Extension of the integrated therapeutic-assistance prevention pathway for the early detection of individuals at risk for MCNT to the prison population and to prison police officers in prisons in the metropolitan cities of Catania, Messina and Palermo | cross-cutting | policy makers; other | adulthood |
| **FRIULI VENEZIA GIULIA (FVG)** | **PP02** | Consolidating *governance* activities | - | policy makers; health professionals | - |
|  |  | Strengthening the health promotion network | - | healthcare professionals | childhood; adolescence; adulthood; working age; women of childbearing age |
|  |  | Training | physical inactivity, hypertension, obesity, smoking | healthcare professionals | childhood; adolescence; adulthood; old age |
|  |  | Further development of the FVG in Motion 10,000 steps of health project | physical inactivity | general population | adolescence; adulthood; working age; women of childbearing age |
|  |  | Activity courses physics | physical inactivity | general population | childhood; adolescence; adulthood; working age; women of childbearing age |
|  |  | Network of health gyms | physical inactivity | other; health professionals | adulthood; old age |
|  |  | Information and communication moments | physical inactivity | general population | childhood; adolescence; adulthood; working age; women of childbearing age |
|  |  | Offering adapted physical activity | physical inactivity, obesity | general population | childhood; adolescence; and adulthood; working age; women of childbearing age |
|  |  | Walking groups | physical inactivity | general population | adulthood; old age |
|  | **OP12** | New edition school catering guidelines | - | policy-maker | - |
|  |  | New edition regional guidelines for kindergartens | - | policy-maker | - |
|  |  | Implementation of a monitoring system of the interventions carried out by the SIAN in support of nutritional quality in mass catering | - | other; health professionals | - |
|  |  | Training of birth pathway operators in accordance with PL 'health promotion in pregnancy and the first 1000 days' | - | healthcare professionals | - |
|  |  | Promoting the dissemination of knowledge and skills of operators involved in the promotion of healthy eating styles on "brief motivational *counselling*" in specific *settings* | - | health professionals; other | - |
|  |  | Dissemination and application of guidelines for the outsourcing of the catering service and support to public bodies for the drafting of tender specifications | - | other | - |
|  |  | Participation in institutional company/regional working tables | - | policy maker | - |
|  |  | Detection of presence/supply of iodized salt at points of sale and in mass catering | - | general population | - |
|  |  | Production of training/information material for the canteen committee, teachers, parents, companies, school catering organisations | - | other | - |
|  |  | Operator training 'nurture your health, at work and at school' | obesity, diabetes, alcohol | health professionals; other | - |
|  |  | Offering nutritional *counselling* for pre-conception and pregnant women or other targets | diabetes, alcohol | health professionals; general population | adulthood; working age |
|  |  | Activate a regional coordination group to liaise with TASIN | - | policy maker | women of childbearing age |
|  |  | Training for official control operators | - | health professionals; other | - |
|  |  | Training for FBO | - | more | - |
|  |  | Publication of information material on food safety issues | - | general population; other | - |
|  |  | Actions to support the social and health system and the third sector for fight against food poverty | - | other | - |
|  | **OP13** | University and health company agreement on physical activity prescription | - | general population | - |
|  |  | Physical activity prescription in the population at increased cardiovascular risk | physical inactivity | general population | adult age (pop with high/very high cv risk factors) |
|  |  | CV screening of working environments | physical inactivity | general population | adulthood; old age |
| **PIEMONTE** | **PP02** | Towards a network to promote an active lifestyle | physical inactivity | policy maker | childhood; adolescence; adulthood; working age; women of childbearing age |
|  |  | Cross-sectoral information and training paths to promote an active lifestyle | cross-cutting | health professionals; other | childhood; adolescence; adulthood; working age; women of childbearing age |
|  |  | *Counselling* to promote an active lifestyle | physical inactivity | healthcare professionals | childhood; adolescence; adulthood; working age; women of childbearing age |
|  |  | Recommend physical activity | physical inactivity | general population | adulthood; old age |
|  |  | Walking groups | physical inactivity | general population | adulthood; old age; working age; women of childbearing age |
|  |  | A map of opportunities for physical and/or sporting activity and active mobility | physical inactivity, social isolation | general population | childhood; adolescence; adulthood; working age; women of childbearing age |
|  |  | Acting on territories through the lens of equity | physical inactivity, social isolation | policy maker | childhood; adolescence; adulthood; working age; women of childbearing age |
|  | **OP12** | Intersectoral' coordination prevention - chronicity | - | policy maker | - |
|  |  | Motivational *counselling* training (national) | cross-cutting | healthcare professionals | childhood; adolescence; adulthood; working age; women of childbearing age |
|  |  | Promoting healthy lifestyles in specific contexts | cross-cutting | policy makers; other | adulthood; old age; working age; women of childbearing age |
|  |  | Health promotion in health care settings for health personnel | - | policy-makers; healthcare professionals | - |
|  |  | Health promotion in deprived territorial contexts | - | policy-maker; other | adulthood; old age; working age |
| **TOSCANA** | **PP02** | Single Regional Programming Act for interventions to promote healthy lifestyles, wellbeing for active and aware communities. | - | policy maker | childhood; adolescence; adulthood; working age; women of childbearing age |
|  |  | Fostering change: brief motivational *counselling* as an operational tool for promoting healthy and sustainable lifestyles | alcohol, smoking, physical activity | healthcare professionals | adulthood; working age |
|  |  | Annual integrated training on AFA (adapted physical activity) for health and non-health professionals | physical inactivity, social isolation | health professionals; other | adulthood; old age; working age |
|  |  | Communicating and informing for smart communities | physical inactivity, social isolation, head injuries | general population | adolescence; adulthood; working age; |
|  |  | Physical activity programmes for individuals with risk factors or who are frail in the region | physical inactivity, social isolation | general population | adulthood; old age; working age |
|  |  | Promotion of physical activity in the Tuscan population of all ages in the presence of risk factors, pathologies or frailty | physical inactivity, social isolation | general population | childhood; adolescence; adulthood; working age; women of childbearing age |
|  |  | Promotion of physical activity in the Tuscan population of all ages | physical inactivity | general population | childhood; adolescence; adulthood; working age; women of childbearing age |
|  |  | Specific alliances with territorial *stakeholders* to create active communities | - | other | adolescence; adulthood; working age; women of childbearing age |
|  |  | Interventions aimed at fostering the acquisition of digital skills in the over-65 population | - | general population | old age |
| **VENETO** | **PP02** | Preparation and dissemination of communication tools for the dissemination of the Active Communities programme | physical inactivity | general population | adolescence; adulthood; old age |
|  |  | Renewal of existing agreements or signing of new agreements for the creation and dissemination of the Active Communities programme | physical inactivity | other | childhood; adolescence; adulthood; old age |
|  |  | Realisation of a training module on brief motivational *counselling* | smoking, alcohol, physical inactivity | healthcare professionals | working age |
|  |  | Realisation of joint training courses for all health and non-health professionals involved in the promotion of motor activity | physical inactivity | health professionals; other | working age |
|  |  | Establishment of the network of active municipalities | - | policy maker | childhood; adolescence; adulthood; old age |
|  |  | Dissemination and capillarisation of exercise prescription and taking care of the patient with chronicity | physical inactivity | health professionals; other | adolescence; adulthood; old age |
|  |  | Adhesion of the Municipality to the Network of Active Municipalities | - | policy maker | childhood; adolescence; adulthood; old age |
|  | **OP11** | Preparation and implementation of a communication plan for the dissemination of MCNT prevention strategies and messages addressed to health professionals | - | healthcare professionals | childhood; adolescence; adulthood; working age; women of childbearing age |
|  |  | Preparation and dissemination of communication tools to disseminate MCNT prevention strategies and messages to the population | - | general population | childhood; adolescence; adulthood; working age; women of childbearing age |
|  |  | Establishment of an inter-sectoral regional operational group for the promotion of healthy lifestyles and prevention of NTMs | - | policy maker | childhood; adolescence; adulthood; working age; women of childbearing age |
|  |  | Realisation of a training module on brief motivational *counselling* | smoking, alcohol, physical inactivity | healthcare professionals | childhood; adolescence; adulthood; working age; women of childbearing age |
|  |  | Realisation of a joint inter-sectoral and inter-professional training module to develop knowledge and skills and disseminate tools for MCNT prevention in the population | - | health professionals; other | childhood; adolescence; adulthood; working age; women of childbearing age |
|  |  | Collective catering in out-of-hospital care facilities for the improvement of nutritional quality | - | other | adulthood; old age |
|  |  | Mapping the Veneto system network for health promotion and MCNT prevention | - | other | childhood; adolescence; adulthood; working age; women of childbearing age |
|  |  | Development and implementation of the Veneto system for health promotion and MCNT prevention | physical inactivity, smoking | policy makers; other | childhood; adolescence; adulthood; working age; women of childbearing age |
|  |  | Implementation of integrated MCNT data visualisation tools and strengthening of tools for early identification of individuals with risk factors | - | general population | childhood; adolescence; adulthood; working age; women of childbearing age |
|  |  | Actions to develop and implement multifactorial MCNT prevention initiatives aimed at the general population | - | healthcare professionals | childhood; adolescence; adulthood; working age; women of childbearing age |
| **MARCHE** | **PP02** | Let's locate health: mapping the supply of motor activity in the Marche region | physical inactivity | general population | adulthood, old age |
|  |  | IMB: network of operators for short-term motivational intervention | smoking, alcohol, physical inactivity | health professionals; other | childhood; adolescence; adulthood; working age; women of childbearing age |
|  |  | Let us do good | physical inactivity | general population | adulthood; old age; working age |
|  |  | Grandparents, grandchildren and the community for health | physical inactivity | general population | childhood; adolescence; old age |
|  |  | City together | physical inactivity, pollution | general population | adulthood, old age |
|  |  | Adapted physical activity/structured physical exercise: implementation of the Marche territorial network | physical inactivity, diabetes | policy-maker | adulthood; old age |
|  |  | Establishment of a regional technical-scientific support group for PP02 | - | other | - |
|  |  | Let's activate health for all: training for operators of day and/or residential centres for the mentally disabled and those with dementia on the promotion of health and healthy lifestyles and with motor activity initiatives. | n.a. (persons already with dementia) | health professionals; other | adulthood |
| **UMBRIA** | **PP02** | Support for programmes to promote physical activitỳ in the population of all age groups | physical inactivity | policy makers; other | childhood; adolescence; adulthood; working age; women of childbearing age |
|  |  | Construction of a targeted and integrated communication campaign to support the physical activity promotion measures to be launched | physical inactivity | general population | childhood; adolescence; adulthood; working age; women of childbearing age |
|  |  | Consolidate/support alliances and *governance* through the activation of an integrated coordination table with the participation of all actors involved in various capacities and the definition of framework agreements with stakeholders at regional level | physical inactivity | policy makers; other | childhood; adolescence; adulthood; working age; women of childbearing age |
|  |  | Training courses to support the programme for the operators involved | physical inactivity | health professionals; other | childhood; adolescence; adulthood; working age; women of childbearing age |
|  | **OP13** | Digital health | - | health professionals; general population | n.a. |
|  |  | Empowerment | - | general population | n.a. |
|  |  | Training | - | healthcare professionals | n.a. |
|  |  | Information | - | general population | n.a. |
| **CAMPANIA** | **PP02** | Establishment of a regional technical group *health equity* audit | - | policy maker | childhood; adolescence; adulthood; old age |
|  |  | Establishment of a regional and corporate monitoring system for the *governance* and monitoring of health promotion programmes/actions/interventions by themes and *settings* and targets | - | policy makers; other | childhood; adolescence; adulthood; old age |
|  |  | Activation of a monitoring and evaluation system of the structured exercise and AFA programmes activated at the facilities of the AFA for Health network established at each ASL. | - | policy makers; other | childhood; adolescence; adulthood; old age |
|  |  | Regional and corporate *governance* actions aimed at reducing the impact of risk factors for active ageing with reduced burden of disease and disability" starting from established experiences (e.g. Cilento Elderly Community) | cross-cutting | other; general population | childhood; adolescence; adulthood; old age |
|  |  | Corporate *governance* actions on health education and promotion for the adoption of healthy lifestyles | cross-cutting | policy makers; other | childhood; adolescence; adulthood; old age |
|  |  | Technical table for the definition of guidelines for the programming of active dementia communities and psychosocial support actions for frailty | - | policy maker |  |
|  |  | Organisation of joint training courses, including those on brief *counselling*, between health and non-health workers involved in the activities̀ of the regional 'afa for health' network | physical inactivity, social isolation | health professionals; other | childhood; adolescence; adulthood; old age |
|  |  | Organisation of short *counselling* training courses for healthcare professionals (including GPs and PLSs) and other non-healthcare stakeholders engaged in activities̀ promoting physical activitỳ in opportunistic settings | smoking, alcohol, physical inactivity | health professionals; other | childhood; adolescence; adulthood; old age |
|  |  | Elaboration of a regional document describing recommended and sustainable practices proposed to local authorities for the creation and enhancement of urban contexts conducive to the promotion of an active lifestyle, accident prevention and the promotion of physical activity by facilitating the inclusion of socio-economically disadvantaged and frail individuals | cross-cutting | policy maker | childhood; adolescence; adulthood; old age |
|  |  | Establishment of an inter-sectoral and multidisciplinary regional coordination table for the creation of a best practice model according to a '*one health*' approach | - | policy maker | childhood; adolescence; adulthood; old age |
|  |  | Implementation of the 'afa territorial network for health | physical inactivity, social isolation | general population | childhood; adolescence; adulthood; old age |
|  |  | Stipulation of AASSLL-municipalities collaboration agreements for the implementation of the objectives set out in the regional document describing the recommended and sustainable practices proposed to local authorities for the creation and enhancement of urban contexts conducive to the promotion of an active lifestyle, accident prevention and the promotion of physical activitỳ facilitating the inclusion of socio-economically disadvantaged and frail̀ individuals | - | policy maker | - |
|  |  | Stipulation of a memorandum of understanding with ANCI Campania for the implementation of actions aimed at fostering the creation of health-friendly contexts in cities, including through the development of sustainable mobility and the creation of green areas and public spaces that are safe, inclusive and accessible also to the elderly | pollution, physical inactivity | other | childhood; adolescence; adulthood; old age |
|  |  | Defining dementia-friendly community building programmes | - | policy maker | - |
|  |  | Collaboration agreements ASL-structures/providers of structured exercise and afa programmes addressed to the population of all age groups with one or more risk factors, or specific pathologies or frailty conditions̀ (elderly), including patients with stabilised MCNT | physical inactivity, social isolation | other | childhood; adolescence; adulthood; old age |
|  |  | Conclusion of regional agreements with CONI, UISP and other associations operating in the promotion of motor and sports activities̀ for the realisation and implementation of the regional 'afa for health' network | physical inactivity, obesity, social isolation | other | childhood; adolescence; adulthood; old age |
|  |  | Awareness-raising actions towards municipalities to reduce the marginalisatioǹ of frail elderly people | social isolation | policy maker | adulthood; old age |
|  |  | Establishment of a joint health - social policy - ANCI technical table for the implementation of programmes/pathways aimed at reducing the impact of risk factors for active ageing and reduced burden of disease and disability | - | policy maker | - |
|  |  | Inform and educate the population on the usefulness of afa and how to participate in programmes | physical inactivity, social isolation | general population | childhood; adolescence; adulthood; old age |
|  |  | Mapping the range of opportunities̀ for motor and sports activities in the area and providing adequate information to citizens on how to access and use them | - | general population | childhood; adolescence; adulthood; old age |
| **CALABRIA** | **PP02** | Short *counselling* training courses for NHS professionals | - | health professionals; other | - |
|  |  | Cross-sectoral training for health professionals (including GPs and PLSs) and other stakeholders | physical inactivity | health professionals; other | adulthood |
|  |  | Promotion of physical activity in the population of all age groups | physical inactivity | general population | childhood; adolescence; adulthood; working age; women of childbearing age |
|  |  | Promotion of physical activity in the population of all age groups with one or more risk factors, specific diseases or frail conditions (elderly) | physical inactivity | general population | childhood; adolescence; adulthood; working age; women of childbearing age |
|  |  | Cooperation agreements with local authorities, institutions, the third sector and sports associations and other stakeholders | - | other | - |
|  |  | Communication and information interventions, targeting both the population and the various stakeholders | physical inactivity | general population; other | childhood; adolescence; adulthood; working age |
| **LAZIO** | **PP02** | Walking groups, urban trekking/green and sustainable paths | physical inactivity | general population | adolescence, adulthood, old age |
|  |  | Communication and monitoring for active communities | physical inactivity | general population | adulthood, old age |
|  |  | Coordination of the Active Communities Programme | physical inactivity | policy makers; other | adulthood, old age |
|  |  | Experimentation and implementation of an AFA, EFA and Otago programme | physical inactivity | policy makers; other | adulthood, old age |
|  |  | Respecting the environment is health (community) | physical inactivity | general population | adolescence, adulthood, old age |
|  |  | Training to support active communities | physical inactivity | health professionals; other | adulthood, old age |
|  |  | Exercise and sporting activity among people with physical, mental, sensory and/or mixed disabilities | physical inactivity | general population | adulthood, old age |
|  | **OP14** | Preventive nutrition | obesity, hypertension | policy makers; other | childhood, adolescence, adulthood, old age |
|  |  | Gaining health in Lazio | smoking, alcohol | policy makers; other | adulthood, old age |
|  |  | Establishment of a network of smoke-free centres (CAFs) | smoke | policy maker | adolescence, adulthood, old age |
|  |  | Experimentation and implementation of an AFA, EFA and Otago programme | physical inactivity | policy makers; other | adulthood, old age |
|  |  | Telemedicine/telehealth and prevention for the integrated management of people at increased risk or with chronic diseases | - | general population | - |
|  |  | Official control of sale/use of iodized salt, allergen management and labelling | - | healthcare professionals | - |
|  |  | Training to support the programme | - | healthcare professionals | - |
|  |  | Programme Coordination | - | policy maker | - |
|  |  | Communication in support of the programme | - | general population | - |
|  |  | Health promotion interventions targeting vulnerable groups | cross-cutting | general population | childhood, adolescence, adulthood, old age |
| **PA TRENTO** | **PP02** | Intersectoral and multidisciplinary coordination group | - | policy maker | adolescence, adulthood, old age |
|  |  | *Health literacy* and population *empowerment* | cross-cutting | general population | adulthood, old age |
|  |  | Programmes to promote physical activitỳ in the population of all age groups̀ with risk factors or frailty conditions̀ | physical inactivity, diabetes, hypertension, social isolation | general population | adulthood, old age |
|  |  | PPDTA: from prevention to cure. Strategies to improve *health literacy* in patients involved in treatment education pathways | cross-cutting | general population | childhood, adolescence, adulthood, old age |
|  |  | Defining the strategy of health-promoting communities | - | policy maker | adolescence, adulthood, old age |
|  |  | Organised risk identification programme aimed at cardiovascular prevention with active calling in 50-year-olds | hypertension, diabetes | policy makers; health professionals | adulthood |
|  |  | Training of GPs, PLS and other practitioners on *counselling* | physical inactivity | healthcare professionals | childhood, adolescence, adulthood, old age |
|  |  |  |  |  |  |
| **PA BOLZANO** | **n.a.** |  | - | - | - |

**AFA**: Attività Fisica Adattata (Adapted Physical Activity); **ANCI**: Associazione Nazionale Comuni Italiani (National Association of Italian Municipalities); **ASL:** Azienda Sanitaria Locale (Local Health Authority); **ATS**/**ASST**: Agenzia di Tutela della Salute/Aziende Socio Sanitaria Territoriali (Health Protection Agencies/Territorial Social Health Agencies); **AUSL**: Azienda Unità Sanitaria Locale (Local Health Unit Company); **BP**: Buone Pratiche (Best Practices); **CAF**: Centro Anti Fumo (Anti-Smoking Centres); **CCM**: Centro nazionale per le prevenzione e il Controllo delle Malattie (National Centre for Disease Prevention and Control); **CONI**: Comitato Olimpico Nazionale Italiano (Italian National Olympic Committee); **DDGG**: Direzioni Generali (General Directorates); **DG**: Direttore Generale (General Director); **EFA**: Esercizio Fisico Adattato (Adapted Physical Exercise); **FVG**: Friuli Venezia Giulia; **IMB** Intervento Motivazionale Breve (Brief Motivational Intervention); **MCNT**: Malattie Croniche Non Trasmissibili (Chronic Noncommunicable Diseases); **MMG:** Medico di Medicina Generale (General Practitioner); **N.A**.: Not Applicable; **OP**: Optional Plan; **PA**: Provincia Autonoma (Autonomous Province); **PDTA**: Percorso Diagnostico-Terapeutico-Assistenziale (Diagnostic Therapeutic Assistential Pathway); **PLS**: Pediatra di Libera Scelta (Paediatrician); **PP**: Predefined Plan; **PPDTA**: Percorso Preventivo-Diagnostico-Terapeutico-Assistenziale (Preventive Diagnostic Therapeutic Assistential Pathway); **RPP**: Regional Prevention Plan; **SIAN**: Servizio di igiene degli alimenti e della nutrizione (Hygene Service for Food and Nutrition); **SPS**: Scuole che Promuovono Salute (Health Promoting Schools); **SSN**: Sistema Sanitario Nazionale (National Health System); **SSR**: Servizio Sanitario Regionale (Regional Health System); **TTM**: Transtheoretical Model of change; **UISP**: Unione Italiana Sport Per tutti (Italian Union Sport for Everyone); **WHP**: Workplace Health Promotion.

**Note:** The translation of the Table is meant to be only for clarification purposes. The official text is in Italian and available in each RPP.

***Table 5*** *-* Dementia prevention bespoke quality checklist disaggregated scores according to RPP.

<https://drive.google.com/drive/folders/1GTXYTKyQmeaBhe3o1HZIere0BTCQmi75?usp=drive_link>

The disaggregated quality checklist scores can be accessed at the provided link.

The first worksheet provides an overview of all assessed RPPs, while the subsequent ones allow the reader to isolate a specific RPP of interest.

***Table 6*** *- List of Planned Programs and Optional Programs addressing the 12 risk factors by RPP*


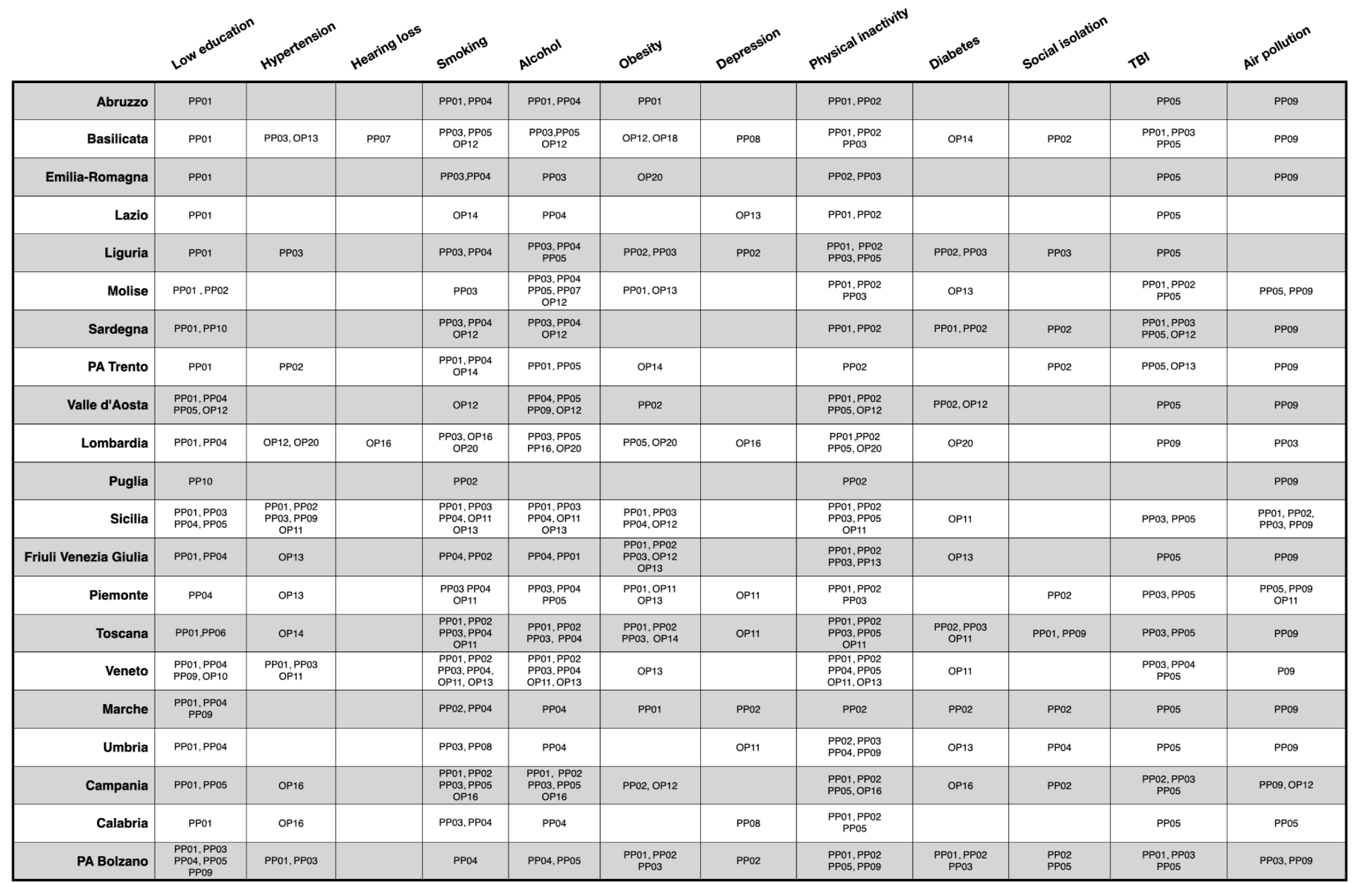


***Figure 1*** *- Planned Programs (PP) and Optional Programs (OP) including dementia-specific (M01OS10) preventive interventions by RPP.*


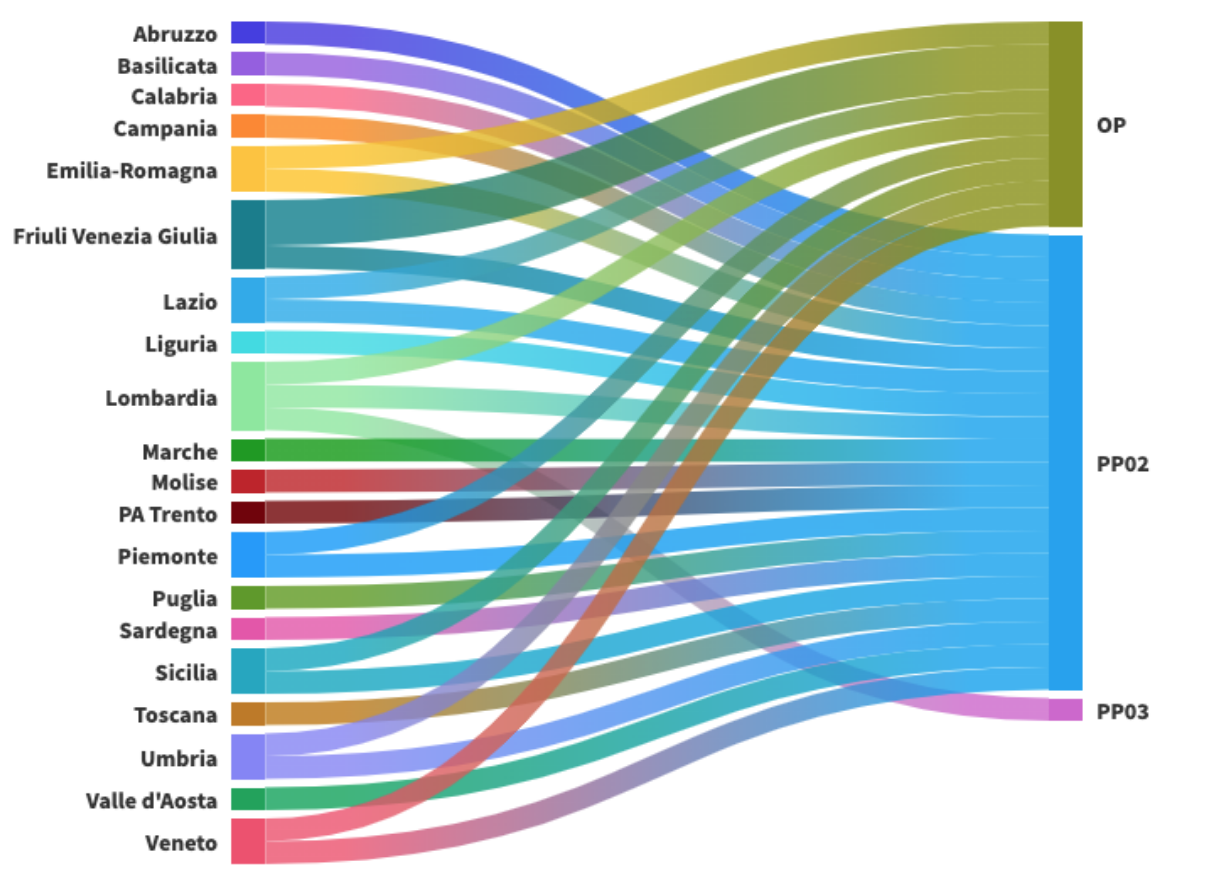


Graphic visualisation of the PPs and OPs adopted by each RPP for the prevention of dementia.

***Figure 2*** *- Risk factors addressed by dementia-specific (M01OS10) preventive interventions by RPP.*


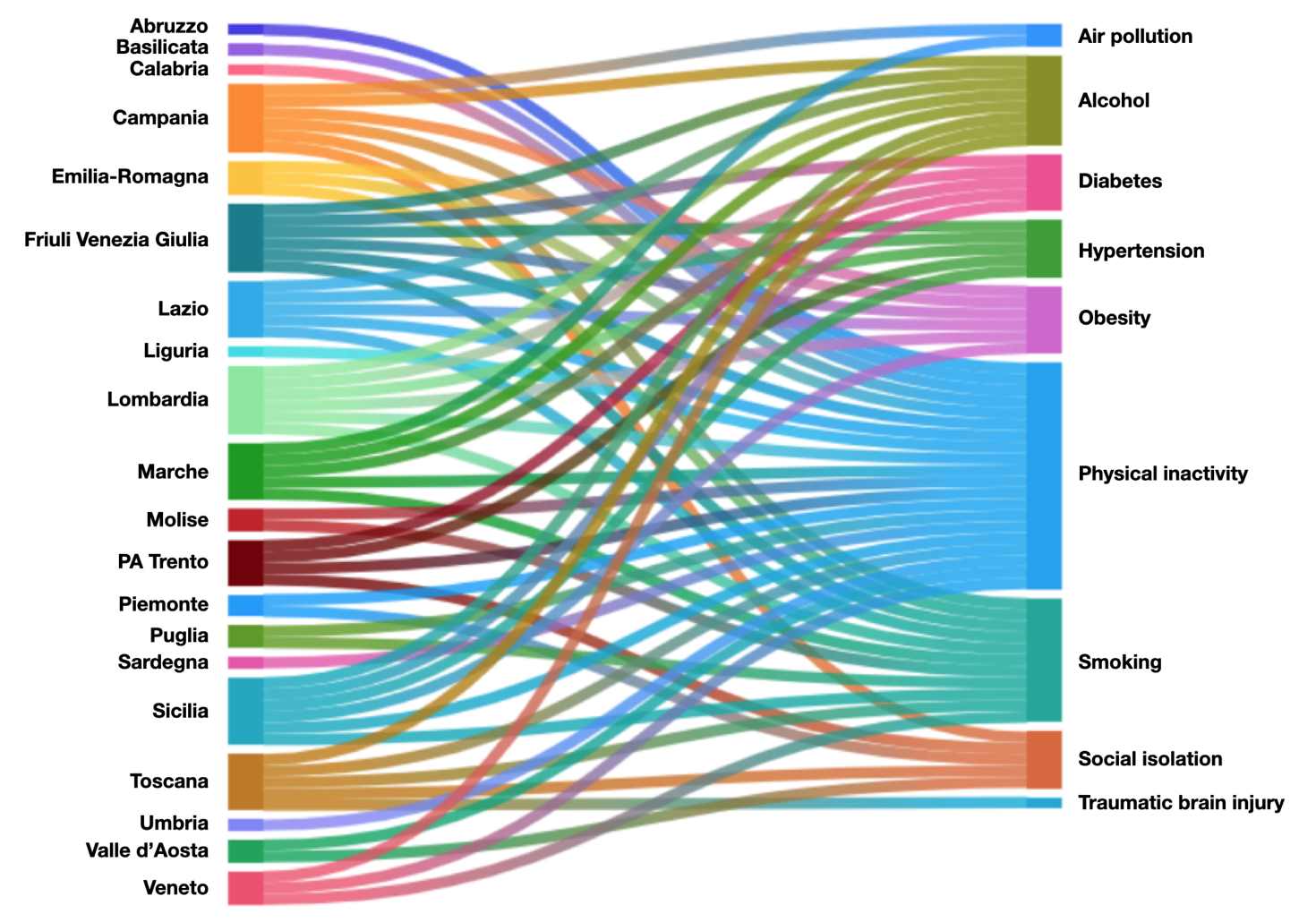


Graphic visualisation of the risk factors addressed by interventions for the prevention of dementia for each RPP.
